# Clinical value of circulating bioactive adrenomedullin for prediction of outcome and hydrocortisone response in sepsis patients—a post-hoc analysis of the HYPRESS trial

**DOI:** 10.1007/s15010-025-02569-x

**Published:** 2025-05-30

**Authors:** Caroline Neumann, Margit Leitner, Frank Bloos, Dorothea Lange, Holger Bogatsch, Djillali Annane, Jerôme Fleuriet, Josef Briegel, Michael Bauer, SepNet Critical Care Trials Group, Michael Bauer, Thorsten Brenner, Patrick Meybohm, Josef Briegel, Markus Weigand, Matthias Gründling, Holger Bogatsch, Markus Loeffler, Michael Kiehntopf, Frank Bloos, Gunnar Elke, Sandra Frank, Melanie Meersch-Dini, Christian Putensen, Achim Kaasch, Stefan Kluge, iRECORDS collaborators, Sara Aly Abdelghany, Maha Aly Khalaf Aly, Mohamed Gamal Elansary, Shereen Mustafa Elgengeehy, Heba Mostafa Elwi, Yasser Sadek Nassar, Rania Yehia Hash, Djillali Annane, Rania Bouneb, Zaineb Chelly-Dagdia, Katy Diallo, Jérome Fleuriet, Henri-Jean Garchon, Stanislas Grassin Delyle, Rahma Hellali, Nicholas Heming, Nicolas Hunzinger, Elodie Lamy, Jihene Mahmoud, Virginie Maxime, Pierre Moine, Camille Roquencourt, Marie Alice Vovy, Karine Zeitouni, Manuela Adling-Ehrhardt, Micheal Bauer, Josef Briegel, Bloos Frank, Sandra Frank, Katharina Habler, Ludwig Hinske, Rainer König, Dorothea Lange, Caroline Neumann, Margit Leitner, Marcus Oswald, Christina Scharf-Janssen, Michael Vogeser, Carlos Flores, Jesús Villar

**Affiliations:** 1 Department of Anaesthesiology and Intensive Care Medicine, Jena University Hospital, Am Klinikum 1, 07747 Jena, Germany; 2 Department of Anaesthesiology, LMU University Hospital, LMU Munich, Marchioninistraße 15, 81377 Munich, Germany; 3 Institute for Medical Informatics, Statistics and Epidemiology (IMISE) and Clinical Trial Centre, Leipzig University, Härtelstraße 16-18, 04107 Leipzig, Germany; 4 IHU PROMETHEUS, Comprehensive Sepsis Centre, Hôpital Raymond Poincaré, Assistance Publique, Hôpitaux de Paris (APHP), 104 Boulevard Raymond Poincaré, 92380 Garches, France

**Keywords:** Sepsis, Septic shock, Hydrocortisone, Bio-adrenomedullin, Predictive enrichment

## Abstract

**Purpose:**

Sepsis requires stratification for host-directed therapies through the discovery of adequate biomarkers enabling prediction of outcomes and treatment responses. Adrenomedullin has previously demonstrated potential for prognostic enrichment. This study aimed to assess associations of bioactive adrenomedullin (bio-ADM) levels at ICU admission and sepsis outcomes and to evaluate the potential of bio-ADM as marker to identify subgroups of patients with moderate disease severity that might benefit from hydrocortisone treatment.

**Methods:**

We used data from the HYPRESS trial (NCT00670254) to investigate, if bio-ADM is useful to predict sepsis outcomes (septic shock, 90- and 180-day mortality) and benefit or harm by hydrocortisone treatment. Optimal cut-offs for outcome predictions were determined by Youden’s index. Logistic regression was used to assess bio-ADM subgroups and treatment interaction.

**Results:**

Bio-ADM levels differed significantly in patients with or without septic shock within 14 days (p = 0.011). While the area under the ROC curve (AUC) was only 0.603 (CI 0.531–0.676), patient subgrouping using bio-ADM levels showed significantly higher cumulative incidence of septic shock within 14 days in the subgroup of patients with bio-ADM levels ≥ 37 pg/mL (p < 0.001). The odds ratio for the development of septic shock in this group was 4.67 (95% CI 1.53, 20.3, p = 0.016). A bio-ADM cut-off of ≥ 136 pg/mL was predictive for 90-day (OR 8.21, 95% CI 2.46–27.9, p < 0.001) and 180-day mortality (OR 4.87, 95% CI 1.49–16.0, p = 0.008). Hydrocortisone therapy did not reduce the incidence of septic shock (OR 1.59, 95% CI 0.37–8.15, p = 0.54), 90-day (OR 1.53, p = 0.23) or 180-day mortality (OR 1.41, p = 0.25), regardless of bio-ADM stratification (interaction term p = 0.58 for septic shock; p = 0.31 for 90-day mortality; p = 0.51 for 180-day mortality).

**Conclusions:**

Whereas bio-ADM levels are associated with sepsis outcomes, our data do not indicate usefulness of the marker to identify patients potentially benefitting from hydrocortisone therapy.

**Supplementary Information:**

The online version contains supplementary material available at 10.1007/s15010-025-02569-x.

## Background

Sepsis is characterised by life-threatening organ dysfunction, following dysregulated host response to infection [1]. Septic shock, the most severe complication of sepsis, is characterised by profound circulatory, cellular, and metabolic abnormalities [1]. Sepsis remains one of the leading causes of morbidity and mortality worldwide, despite appropriate anti-infective treatment, removal of infectious foci, and organ support [2]. Sepsis encompasses a myriad of subphenotypes related to different types of infection, variable host responses from exaggerated inflammation to immune suppression and involves a broad diversity of systems, e.g. neuro-endocrine, immune, complement, coagulation, endothelium and microbiome [3]. Biomarkers may help deciphering molecular pathways of specific subphenotypes [4]. They may improve early identification of patients at risk of worsening (prognostic enrichment), and those who may benefit or be harmed by a given therapy (predictive enrichment) [5, 6]. The administration of corticosteroids is one of the few immunomodulatory treatments for selected patients with septic shock [7] despite controversial results [8–13], side effects, and corticosteroid resistance [14]. Next to sepsis and based on current evidence [15–17], corticosteroids should be administered to adult patients hospitalized with severe bacterial community-acquired pneumonia [18].

Sepsis induces haemodynamic instability with often therapy-refractory systemic vasoplegia and endothelial dysfunction, leading to capillary leakage. This often results in impaired perfusion of organs, causing organ dysfunction or failure. In addition to an adequate volume therapy, the administration of vasopressors is recommended to maintain organ perfusion and microcirculation.

Adrenomedullin (ADM) contributes to the regulation of endothelial function and vascular integrity [19–21] by stabilising endothelial barrier but also has multiple physiological effects on various organs (such as the kidneys and the lungs) and on steroid synthesis, thus participating in a complex homeostatic response. As a regulator of renal function, ADM leads to an increase in renal blood flow and glomerular filtration rate [22, 23]. In particular its role as an inhibitor of cortisol synthesis and secretion [24] and the reversal of this effect after corticosteroid treatment [25] make it an interesting candidate biomarker for the prediction of individual patient responses to hydrocortisone (HC) therapy and may prevent harm to patients by unnecessary exposure to this therapy. Preclinical evidence investigating the biological interaction of steroids and bio-ADM in sepsis patients is currently lacking. Numerous studies addressed the effectiveness of HC treatment alone [9] or in combination with fludrocortisone (FC) in septic shock [8]. The two largest trials in patients with septic shock (APROCCHS and ADRENAL) reported earlier shock reversal and liberation from mechanical ventilation upon corticosteroid treatment but conflicting results on mortality [8, 13]. A meta-analysis of septic shock trials found no significant effect on the relative risk for 90-day mortality in HC versus usual care [26]. Data from the ADRENAL trial have also shown no reduction in 90-day and 6-months mortality in septic shock patients treated with hydrocortisone compared to placebo [13, 27]. Dequin and colleagues [15] demonstrated survival benefits at 28-day from hydrocortisone in ICU patients with severe community-acquired pneumonia. In contrast, the “Hydrocortisone for Prevention of Septic Shock” (HYPRESS) study (NCT00670254) found no significant effect of hydrocortisone, as compared to placebo, on the onset of septic shock or mortality in 353 adult sepsis patients with moderate disease severity [10].

The current post-hoc analysis of the HYPRESS trial aimed at assessing the previously reported prognostic value of circulating bio-ADM levels to predict sepsis outcomes [28–30] and the potential for predictive enrichment regarding the patient’s response to hydrocortisone therapy.

## Materials and methods

### Study design and study population

The methods of the HYPRESS trial have been detailed previously (NCT00670254) [10]. Briefly, adults with evidence of infection, at least two criteria of the systemic inflammatory response syndrome (SIRS) [31] and evidence of vital organ dysfunction for a maximum of 48 h were included. Patients with septic shock, defined as sepsis-induced hypotension despite adequate volume resuscitation for more than 4 h, were excluded [32]. Hydrocortisone was administered as an intravenous bolus of 50 mg, followed by a 24-h continuous infusion of 200 mg per day for 5 days, 100 mg on days 6 and 7, 50 mg on days 8 and 9, and 25 mg on days 10 and 11 [10]. In this post hoc study, we investigated the potential of bio-ADM to predict the occurrence of septic shock within 14 days (primary endpoint). Secondary endpoints included all-cause 90-day and 180-day mortality rates, ICU and hospital length of stay, number of days (up to day 28) of dependency on mechanical ventilation or RRT, and the difference in SOFA score between day 28 and baseline. We further assessed if bio-ADM levels could be used for predictive enrichment of patient sub-populations that could benefit (e.g. in terms of shock reversal, reduction of mortality) or be harmed (e.g. increased incidence of shock and mortality rates) by hydrocortisone treatment.

### Biomarker measurement

Citrate plasma samples were taken upon inclusion in the HYPRESS trial and stored at -80°C Bio-ADM was measured using the immunoluminometric sphingotest® assay (SphingoTec GmbH, Hennigsdorf, Germany) as previously described [33]. Reference ranges for bio-ADM, derived from a community-based cohort of 5060 subjects, are of 8–30 pg/mL (95% CI 26–34 pg/mL) [34]. The laboratories performing the biomarker measurements were blinded to clinical and demographic patient data.

### Statistical analysis

All statistical analyses were performed using R version 4.2.0 (https://www.r-project.org/). Summary statistics were compiled with the package gtsummary [35] and values are expressed as medians and interquartile ranges (IQR), or counts and percentages, as appropriate. Analysis and data visualization of incidence of septic shock and survival was done using the R packages survival [36], tidycmprsk [37], ggsurvfit [38] and survminer [39]. After confirming that baseline data of the reduced cohort are equivalent to the complete dataset, the performance of bio-ADM for prediction of primary and secondary endpoints was assessed using Receiver operating characteristic (ROC) curve analysis.

ROC curves, including determination of optimal cut-offs for the prediction of defined endpoints using Youden’s index, were calculated as implemented in the R package OptimalCutpoints [40]. All analyses were performed on data from the intention-to-treat population.

For the primary endpoint, incidence of septic shock within 14 days, and the secondary endpoints 90- and 180-day mortality, logistic regression was applied to assess differences between the determined bio-ADM subgroups and the interaction with treatment arms. The models included the treatment arm, the indicator for the bio-ADM group, and the interaction term of the two and were adjusted for age, sex and renal dysfunction. For the primary endpoint, we also assessed the impact of these factors on model performance using the likelihood ratio test. Further differences in secondary outcomes, as indicated by the Kruskal–Wallis rank sum test, were analysed by adding Dunn’s post-hoc test with Bonferroni correction.

## Results

### Patient baseline characteristics of the whole cohort by treatment arm

Of 380 randomized patients, 353 were assigned to the intent-to-treat (ITT). After dropping out patients with missing consent for post-hoc analysis or for whom no blood samples were available, we analysed data from 317 patients of the ITT population (65% male, median age 68 [IQR 55–75] years, median body mass index 26.1 [IQR 23.4–29.7], median SOFA score of 6 [IQR 5–8]) (Suppl. Table 1). Baseline data of the reduced cohort did not significantly differ from the complete dataset of the original HYPRESS population [10]. There were no significant differences between hydrocortisone and placebo in the proportion of patients who developed septic shock within 14 days from randomization (Suppl. Table 2, Suppl. Fig. 1). The majority thereof suffered from early septic shock, within 3 days of randomization (Suppl. Fig. 1).

### Patient baseline characteristics by treatment arms and bio-ADM stratification

Bio-ADM levels differed significantly between patients with (median 63.0 pg/mL, IQR 45.1–95.7) or without (median 52.1 pg/mL, IQR 31.1–85.9) developing septic shock within 14 days (two sample t-test p = 0.004, Suppl. Fig. 2). The area under the ROC curve (AUC) for the prediction of the primary endpoint, septic shock, by bio-ADM levels was 0.603 (0.531, 0.676) with an optimal bio-ADM cut-off level of 37 pg/mL (see below and Table 3).

**Fig. 1:**
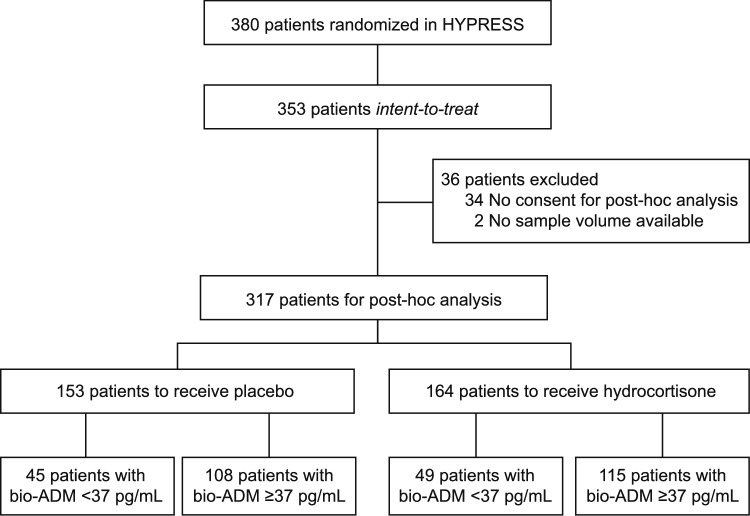
Flow diagram of the HYPRESS patient population analysed in this post-hoc analysis stratified by bio-ADM levels

223 of the 317 patients (70.3%) had bio-ADM levels greater or equal 37 pg/mL, 115 of them (51.6%) were treated with hydrocortisone (see Fig. 1).

The baseline characteristics of the treatment arms stratified by bio-ADM differ with respect to age, SOFA and APACHE score (Table 1). Patients with bio-ADM levels ≥ 37 pg/mL showed lower maximum mean arterial pressure (MAP) and more renal dysfunction(expressed in lower urine output within 24 h after admission and higher serum creatinine levels) 24 h before study inclusion. Patients in the high bio-ADM group (≥ 37 pg/mL) showed higher values for inflammatory markers (CRP and PCT) compared to those in the low bio-ADM group (< 37 pg/mL). In the placebo group, 87% of patients with bio-ADM levels below 37 pg/mL showed signs of arterial hypoxaemia at baseline in contrast to only 67% of patients with high levels. In the high bio-ADM group there was no difference in arterial hypoxaemia between treatment and placebo group. Related to the focus of infection, high bio-ADM levels were more frequent in cases with genitourinary infections, whereas in pneumonia, the percentage of patients in the low bio-ADM group was higher (Table 1)*.* Given the differences in disease severity indicators and age, we further examined associations of bio-ADM levels and clinical risk factors. Whereas age did not correlate with bio-ADM levels (Pearson correlation coefficient r = -0.02, p = 0.7), female patients showed significantly higher baseline bio-ADM levels (median 65.1 pg/mL, IQR 38.0–100.5 pg/mL) than male patients (median 51.7, IQR 32.4–75.3 pg/mL; Wilcoxon rank sum test p = 0.022). Moreover, baseline bio-ADM levels differed in patients with renal dysfunction (as defined in Table 1), with median levels of 47.4 pg/mL (IQR 29.6–69.0) without and 72.4 pg/mL (IQR 47.9–115.7) with impaired kidney function.

**Table 1:** Baseline characteristics of the patient population by bio-ADM groups below or above the 37 pg/mL cut-off and treatment arm

Characteristic	N	Overall		Placebo		Hydrocortisone	
		< 37 pg/mL, N = 94^a^	≥ 37 pg/mL, N = 223^a^	< 37 pg/mL, N = 45^a^	≥ 37 pg/mL, N = 108^a^	< 37 pg/mL, N = 49^a^	≥ 37 pg/mL, N = 115^a^
Age (years)	317	62.0 (49.5–72.8)	71.0 (60.0–76.0)	61.0 (47.0–71.0)	70.5 (63.0–76.0)	65.0 (51.0–74.0)	71.0 (56.5–77.0)
Male sex	317	67/94 (71%)	138/223 (62%)	31/45 (69%)	65/108 (60%)	36/49 (73%)	73/115 (63%)
Body Mass Index	316	25.0 (22.9–28.2)	26.2 (23.9–30.4)	26.6 (23.4–28.4)	26.3 (23.7–30.8)	24.5 (22.0–27.5)	25.8 (23.9–29.9)
SOFA score	267	5.0 (4.0–7.0)	6.0 (5.0–8.0)	5.0 (4.0–7.0)	6.0 (5.0–8.0)	5.0 (4.0–7.0)	7.0 (5.0–8.0)
Sub-SOFA respiratory system	299	3.0 (2.0–3.0)	3.0 (2.0–3.0)	3.0 (2.0–3.0)	3.0 (2.0–3.0)	3.0 (2.0–3.0)	3.0 (2.0–3.0)
Sub-SOFA nervous system	317	0.0 (0.0–1.0)	0.0 (0.0–1.0)	0.0 (0.0–1.0)	0.0 (0.0–1.0)	0.0 (0.0–1.0)	0.0 (0.0–1.0)
Sub-SOFA cardiovascular system	310	1.0 (0.0–1.0)	1.0 (0.0 – 1.0)	1.0 (0.0 – 1.0)	1.0 (0.0 – 1.0)	1.0 (0.0 – 1.0)	1.0 (0.0 – 1.0)
Sub-SOFA liver	290	0.0 (0.0–0.0)	0.0 (0.0–1.0)	0.0 (0.0–0.0)	0.0 (0.0–0.5)	0.0 (0.0–0.0)	0.0 (0.0–1.0)
Sub-SOFA coagulation	316	0.0 (0.0–0.0)	0.0 (0.0–1.0)	0.0 (0.0–0.0)	0.0 (0.0–1.0)	0.0 (0.0–0.0)	0.0 (0.0–1.0)
Sub-SOFA kidneys	315	0.0 (0.0–1.0)	1.0 (0.0–2.0)	0.0 (0.0–1.0)	1.0 (0.0–2.0)	0.0 (0.0–1.0)	1.0 (0.0–2.0)
APACHE score	317	16.0 (12.0–21.0)	19.0 (15.0–22.5)	16.0 (13.0–21.0)	19.0 (16.0–22.0)	16.0 (12.0–21.0)	18.0 (15.0–23.0)
SIRS criteria							
Temperature ≤ 36°C or ≥ 38°C	317	74/94 (79%)	166/223 (74%)	35/45 (78%)	86/108 (80%)	39/49 (80%)	80/115 (70%)
Heart rate ≥ 90 beats/min	317	87/94 (93%)	205/223 (92%)	43/45 (96%)	103/108 (95%)	44/49 (90%)	102/115 (89%)
Tachypnea, hypocapnia, or mechanical ventilation	316	83/94 (88%)	198/222 (89%)	43/45 (96%)	97/108 (90%)	40/49 (82%)	101/114 (89%)
Leukocytosis, leukopenia, or left shift	317	68/94 (72%)	165/223 (74%)	33/45 (73%)	79/108 (73%)	35/49 (71%)	86/115 (75%)
Physiological variables^b^							
Max. heart rate (1/min)	316	117.0 (102.0–126.0)	115.0 (101.0–130.0)	114.0 (100.0–126.0)	116.5 (103.0–129.3)	118.5 (107.3–126.0)	111.0 (100.0–130.0)
Min. MAP (mmHg)	299	67.0 (61.0–78.0)	64.0 (59.0–73.0)	67.0 (60.0–77.3)	65.0 (60.0–74.0)	67.5 (61.8–78.0)	64.0 (58.0–72.3)
Max. respiratory rate (1/min)	270	29.0 (25.0–34.0)	28.0 (24.0–33.0)	29.0 (24.5–34.0)	29.0 (24.0–33.0)	30.0 (25.3–33.8)	27.0 (23.3–33.0)
Urinary excretion (ml/24h)	307	2,340.0 (1,348.0–3,290.0)	1,705.0 (960.0–2,797.5)	2,084.5 (1,363.3–3,197.5)	1,814.5 (1,013.0–3,317.3)	2,520.0 (1,324.0–3,350.0)	1,630.0 (844.5–2,482.5)
Min. PaO_2_/FiO_2_ ratio (mmHg)	289	171.0 (111.0–222.0)	192.5 (138.6–258.3)	168.0 (128.0–207.0)	190.0 (132.5–244.0)	188.0 (107.5–230.8)	202.0 (150.0–271.0)
Organ dysfunction							
Acute encephalopathy^c^	315	17/92 (18%)	60/223 (27%)	8/45 (18%)	31/108 (29%)	9/47 (19%)	29/115 (25%)
Renal dysfunction^d^	317	19/94 (20%)	105/223 (47%)	12/45 (27%)	52/108 (48%)	7/49 (14%)	53/115 (46%)
Coagulopathy^e^	317	13/94 (14%)	43/223 (19%)	5/45 (11%)	18/108 (17%)	8/49 (16%)	25/115 (22%)
Arterial hypoxaemia^f^	316	72/94 (77%)	142/222 (64%)	39/45 (87%)	65/107 (61%)	33/49 (67%)	77/115 (67%)
Microcirculatory dysfunction^g^	316	22/94 (23%)	83/222 (37%)	9/45 (20%)	39/108 (36%)	13/49 (27%)	44/114 (39%)
Laboratory values							
Leukocytes min. [G/l]	314	13.5 (8.1–17.0)	12.0 (7.1–18.0)	14.0 (8.0–19.0)	11.6 (7.1–16.9)	13.0 (8.7–15.5)	12.0 (7.7–18.1)
Platelets, min. [G/l]	316	218.5 (155.8–301.0)	186.0 (128.3–272.8)	207.0 (152.0–354.0)	190.0 (138.3–278.3)	222.0 (171.0–298.0)	177.0 (120.5–271.5)
Max. bilirubin (µmol/l)	259	10.0 (7.0–17.0)	14.0 (9.0–22.0)	10.0 (7.0–17.0)	14.0 (9.0–21.0)	10.5 (7.0–17.5)	13.5 (7.8–22.0)
Max. serum creatinine (µmol/l)	249	88.0 (61.4–118.0)	133.0 (88.0–224.0)	88.0 (61.4–130.5)	159.1 (106.0–261.0)	88.0 (61.0–113.5)	123.0 (75.0–218.5)
Max. urea (mmol/l)	303	6.0 (4.0–10.0)	11.0 (6.8–17.0)	7.0 (3.7–10.0)	11.6 (7.0–17.9)	5.7 (4.0–9.6)	11.0 (6.2–16.5)
CRP level [mg/l]	240	171.0 (108.8–228.5)	242.5 (165.8–318.3)	189.5 (127.3–230.0)	243.5 (168.3–310.3)	160.0 (102.8–219.5)	232.5 (166.0–318.8)
PCT level [ng/ml]	227	1.0 (0.0–4.0)	3.5 (1.0–13.0)	1.0 (0.0–4.0)	3.0 (1.0–9.0)	1.0 (0.0–4.0)	5.0 (1.0–18.3)
Max. lactate at baseline [mmol/l]	307	1.5 (1.1–2.4)	1.8 (1.3–2.7)	1.2 (1.1–2.1)	1.8 (1.3–2.8)	1.6 (1.2–2.5)	1.9 (1.3–2.6)
bio-ADM [pg/mL]	317	26.4 (17.4–31.1)	70.1 (52.2–109.7)	27.2 (18.5–31.6)	70.4 (53.6–97.9)	25.7 (15.0–30.6)	70.0 (51.1–120.1)
Focus of infection	317						
Pneumonia		48/94 (51%)	74/223 (33%)	26/45 (58%)	43/108 (40%)	22/49 (45%)	31/115 (27%)
Other infections of upper or lower airways		7/94 (7.4%)	10/223 (4.5%)	2/45 (4.4%)	7/108 (6.5%)	5/49 (10%)	3/115 (2.6%)
Thoracic (Empyem/Mediastinitis)		4/94 (4.3%)	4/223 (1.8%)	1/45 (2.2%)	0/108 (0%)	3/49 (6.1%)	4/115 (3.5%)
Gastrointestinal		2/94 (2.1%)	12/223 (5.4%)	0/45 (0%)	5/108 (4.6%)	2/49 (4.1%)	7/115 (6.1%)
Primary bacteremia		0/94 (0%)	4/223 (1.8%)	0/45 (0%)	2/108 (1.9%)	0/49 (0%)	2/115 (1.7%)
Catheter-associated infection		1/94 (1.1%)	7/223 (3.1%)	0/45 (0%)	2/108 (1.9%)	1/49 (2.0%)	5/115 (4.3%)
Bones/soft tissues		8/94 (8.5%)	18/223 (8.1%)	3/45 (6.7%)	8/108 (7.4%)	5/49 (10%)	10/115 (8.7%)
Surgical wound infection		1/94 (1.1%)	5/223 (2.2%)	0/45 (0%)	3/108 (2.8%)	1/49 (2.0%)	2/115 (1.7%)
Intra-abdominal		19/94 (20%)	40/223 (18%)	8/45 (18%)	16/108 (15%)	11/49 (22%)	24/115 (21%)
Central nervous system		1/94 (1.1%)	2/223 (0.9%)	0/45 (0%)	1/108 (0.9%)	1/49 (2.0%)	1/115 (0.9%)
Genitourinary		2/94 (2.1%)	36/223 (16%)	2/45 (4.4%)	15/108 (14%)	0/49 (0%)	21/115 (18%)
Unknown		12/94 (13%)	41/223 (18%)	5/45 (11%)	22/108 (20%)	7/49 (14%)	19/115 (17%)

*SOFA* Sequential Organ Failure Assessment, *APACHE* Acute Physiology and Chronic Health Evaluation, *SAPS* simplified acute physiology score, *SIRS* systemic inflammatory response syndrome, *MAP* mean arterial pressure, *PCT* procalcitonin, *CRP* C-reactive protein, *bio-ADM* bioactive adrenomedullin, *ICU* intensive care unit

^a^Median (25%-75%); n/N (%)

^b^24 h time interval before study inclusion

^c^Reduced vigilance, restlessness, disorientation, delirium, unaffected by psychotropic drugs

^d^Oliguria: < 0.5 mL/kg/h for at least 2 h despite adequate volume replacement and/or creatinine increase ≥ 2 times above the reference value of the respective laboratory and/or renal replacement therapy

^e^Thrombocytopenia ≤ 100,000/µL or more than a 30% decrease from baseline within 24 h (not caused by bleeding or immunological factors)

^f^PaO_2_ ≤ 75 mmHg [≤ 10 kPa] under room air or a PaO_2_/FiO_2_ ≤ 250 mmHg [≤ 33 kPa] under oxygen administration (Not due to pre-existing heart or lung disease)

^g^Lactate > 1.5 times the upper reference range and/or base deficit ≥ 5 mmol/L and/or metabolic acidosis with pH < 7.3 and/or impaired capillary reperfusion and/or marbling and/or significant edema in capillary leak syndrome

### Determination of bio-ADM cut-off levels for outcome prediction

**Fig. 2:**
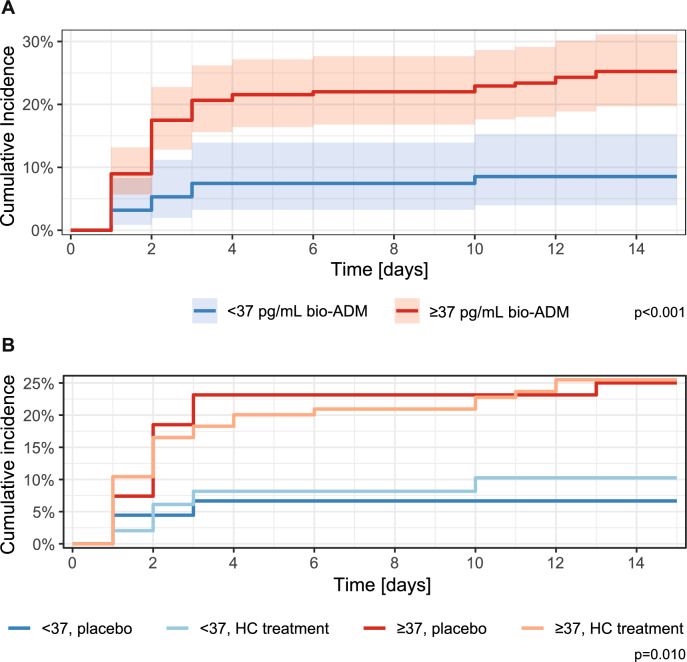
Cumulative incidence of septic shock within 14 days **A** by bio-ADM groups < 37 and ≥ 37 pg/mL and **B** by bio-ADM groups < 37 and ≥ 37 pg/mL and by treatment groups

As outlined above, the optimal bio-ADM cut-off level for the prediction of the primary endpoint, septic shock, was determined to be 37 pg/mL using Youden’s index. Cumulative incidence of septic shock within 14-days differed significantly between bio-ADM groups below or above the determined cut-off of 37 pg/mL (p < 0.001, Fig. 2A, Suppl. Fig. 3), whereas no significant differences were found between placebo and HC treatment arms (Fig. 2B). Logistic regression for this endpoint showed an odds ratio (OR) of 4.67 for development of septic shock in the group with bio-ADM levels ≥ 37pg/mL (p = 0.016), but no significant differences connected to HC treatment or interaction of bio-ADM and treatment (Suppl. Table 3). Given the differences in baseline data outlined above, we further calculated a model adjusted for age, sex and renal dysfunction, in which bio-ADM levels ≥ 37pg/mL remained predictive of septic shock within 14 days (OR 4.40, p = 0.022; Suppl. Table 4). Model comparison using the likelihood ratio test confirmed significant association of bio-ADM levels ≥ 37pg/mL with shock development (p = 0.013).

Except for the significantly higher incidence of septic shock in patients with baseline bio-ADM levels ≥ 37 pg/mL, there were no differences using this cut-off between the further outcomes investigated (mortality until day 28, 90, and 180, length of ICU and hospital stay, days with mechanical ventilation, RRT, and in delta SOFA score; Table 2), whereas bio-ADM levels differed between survivors and non-survivors (Suppl. Fig. 4).

**Table 2:** Main outcomes of the patient population by bio-ADM cut-off 37 pg/mL and treatment arm

Outcomes	N	Overall		Placebo		Hydrocortisone	
		< 37, N = 94^a^	≥ 37, N = 223^a^	< 37, N = 45^a^	≥ 37, N = 108^a^	< 37, N = 49^a^	≥ 37, N = 115^a^
Septic shock until day 14	317	8/94 (8.5%)	56/223 (25%)	3/45 (6.7%)	27/108 (25%)	5/49 (10%)	29/115 (25%)
28-day mortality	302	6/91 (6.6%)	7/211 (3.3%)	3/44 (6.8%)	2/103 (1.9%)	3/47 (6.4%)	5/108 (4.6%)
90-day mortality	284	6/84 (7.1%)	22/200 (11%)	2/40 (5.0%)	12/100 (12%)	4/44 (9.1%)	10/100 (10%)
180-day mortality	308	19/91 (21%)	56/217 (26%)	9/43 (21%)	23/106 (22%)	10/48 (21%)	33/111 (30%)
ICU length of stay [d]	315	7.0 (5.0–11.0)	7.0 (4.0–16.0)	7.0 (5.0–10.0)	8.0 (5.0–18.0)	8.0 (5.0–11.3)	7.0 (4.0–14.8)
Hospital length of stay [d]	315	20.0 (12.0–36.0)	24.0 (14.3–47.0)	18.0 (11.0–25.0)	23.0 (14.0–47.8)	21.5 (14.3–47.8)	24.0 (15.0–47.0)
Days with mech ventilation until D28 (on ICU)	315	1.0 (0.0–5.0)	1.0 (0.0–6.0)	2.0 (0.0–4.0)	2.0 (0.0–7.0)	1.0 (0.0–5.0)	0.5 (0.0–5.0)
Δ SOFA	246	0.0 (– 1.0 to 2.0)	1.0 (– 1.0 to 3.0)	0.0 (– 1.8 to 2.8)	1.0 (– 1.0 to 3.0)	0.0 (– 0.3 to 1.3)	1.0 (– 1.0 to 3.0)
Days with RRT until D28 (on ICU)^b^	315	0.4 (2.1)	0.9 (3.0)	0.5 (2.2)	0.8 (3.0)	0.4 (2.0)	0.9 (2.9)

*ICU* intensive care unit, *RRT* renal replacement therapy

^a^Median (25%-75%); n/N (%)

^b^Mean (sd); Median and interquartile range are 0 in all groups

As a consequence, additional cut-off levels for the prediction of 90- and 180-day mortality by ADM levels were calculated (Table 3). No useful and reliable cut-off could be determined for prediction of 28-day mortality.

**Table 3:** Optimal bio-ADM cut-offs for prediction of endpoints septic shock, 90- and 180-day mortality, as determined by Youden’s index

Endpoint	bio-ADM cut-off [pg/mL]	AUC (CI)	Sensitivity	Specificity	NPV	PPV	Positive DLR	Negative DLR
Septic shock	37	0.603 (0.531, 0.676)	0.875	0.340	0.914	0.251	1.326	0.368
90-day mortality	136	0.585 (0.495, 0.675)	0.273	0.918	0.855	0.417	3.338	0.792
180-day mortality	73.1	0.585 (0.506, 0.663)	0.453	0.721	0.804	0.343	1.625	0.758

*AUC* area under the ROC curve, *DLR* diagnostic likelihood ratio, *NPV* negative predictive value, *PPV* positive predictive value

### Prediction of death by bio-ADM levels

**Fig. 3:**
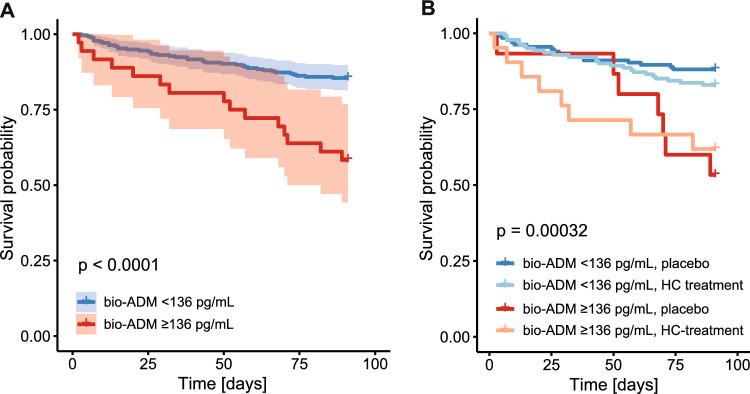
T90-day mortality **A** by bio-ADM groups < 136 and ≥ 136 pg/mL and **B** by bio-ADM groups < 136 and ≥ 136 pg/mL and HC treatment

**Fig. 4:**
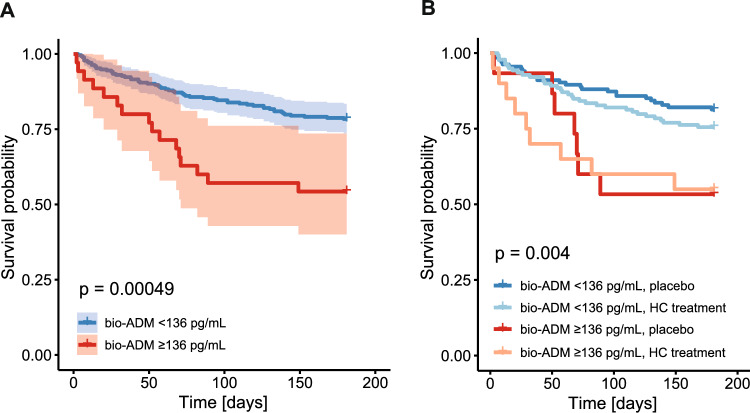
T180-day mortality **A** by bio-ADM groups < 136 and ≥ 136 pg/mL and **B** by bio-ADM groups < 136 and ≥ 136 pg/mL and HC treatment

No significant differences were detected in 28-day, 90-day or 180-day survival between treatment arms (Suppl. Table 2). As the optimal bio-ADM cut-off determined for the primary outcome, septic shock, was not informative for any other outcomes, we additionally calculated cut-off values for the prediction of 90- and 180-day mortality (136 pg/mL and 73.1 pg/mL, respectively; see Table 3). Data for the 73.1 pg/mL cut-off are shown in Suppl. Fig. 5 and Suppl. Tables 5 and 6. Overall, the bio-ADM cut-off of 136 pg/mL performed better for prediction of mortality (Suppl. Table 7 and 8, Fig. 3, Fig. 4). Mortality was significantly lower in patients with baseline bio-ADM levels < 136 pg/mL (Suppl. Fig. 6).

For 90-day mortality, adjusted logistic regression indicated an OR of 8.21 in the high bio-ADM group (p < 0.001) and an OR of 4.87 (p = 0.008) for 180-day mortality. Age significantly influenced mortality, but with an OR of only 1.03 (p = 0.012) for 90-day mortality and 1.04 (p < 0.001) for 180-day mortality. Again, no differences in treatment effects could be detected in the different bio-ADM groups (Suppl. Tables 7, 8).

We further analysed differences in baseline statistics and all other secondary outcomes using the bio-ADM cut-off 136 pg/mL (Suppl. Tables 9 and 10). Baseline characteristics were largely in line with the results received patients grouped by the 37 pg/mL bio-ADM cut-off. In contrast to the 37 pg/mL cut-off, patients categorized by bio-ADM levels below or above 136 pg/mL displayed significant differences in microcirculatory dysfunction (Suppl. Table 9), present in 28% and 31% of the patients in the low bio-ADM group vs. 60% and 62% of the patients of the high bio-ADM group (placebo and HC-treated, respectively, for both groups). Using this biomarker-based patient categorization, significant differences in ICU and hospital LOS became apparent, as well as in delta SOFA score and days with RRT until day 28 (**Suppl. Table 10**).

### Influence of focus of infection on bio-ADM levels

**Fig. 5:**
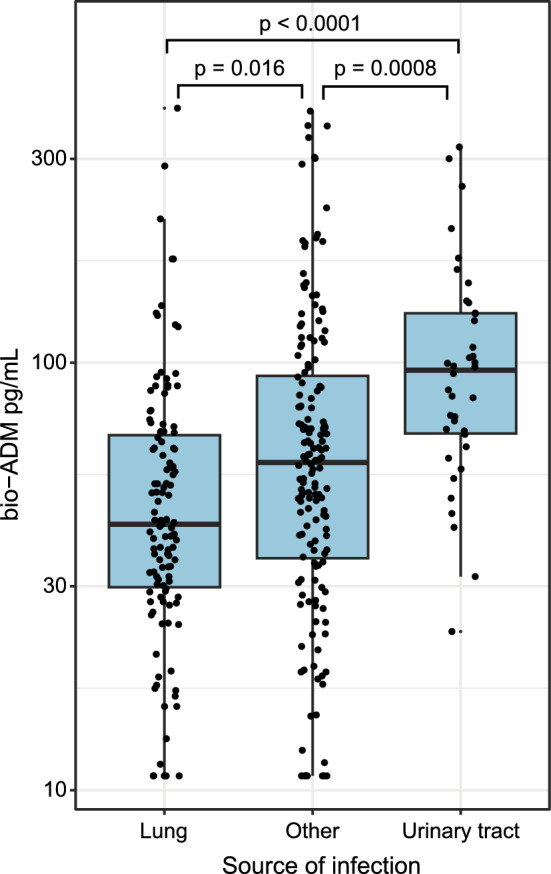
Bio-ADM levels by source of infection. Bio-ADM levels differ significantly in lung infections (p = 0.016) and urinary tract infections (p = 0.0008) compared to other infectious foci. Adjusted p-values are given as determined by ANOVA of log-transformed bio-ADM values and paired t-test using Bonferroni correction

The lungs were the organs most often affected by infection. 122 out of 317 patients (38%) had pneumonia upon admission. In patients with pneumonia, bio-ADM levels were significantly (p = 0.016) lower at admission compared to patients with other sources of infection (Fig. 5). Differences were even more pronounced compared to urinary tract infections (p = 3.9e-07). In patients with genitourinary infection (n = 38), bio-ADM levels were higher compared to other infection sources (p-value = 0.0008) (Fig. 5).

**Fig. 6:**
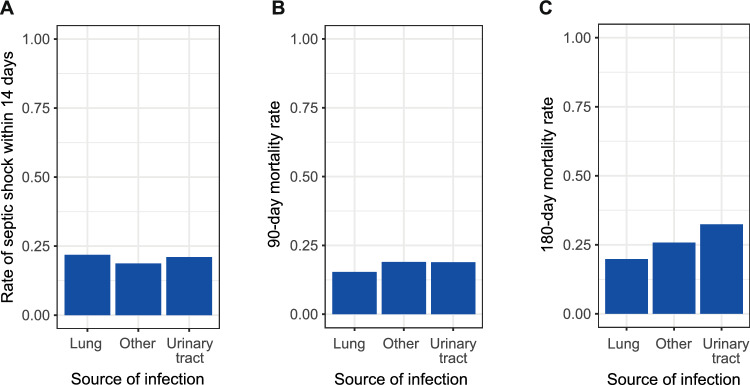
Main outcomes by focus of infection (lung n = 119; other n = 160; urinary tract n = 38. **A** Septic shock within 14 days (Pearson's Chi-squared test p = 0.8), **B** 90-day mortality (p = 0.7) and **C** 180-day mortality (p = 0.2)

Despite the striking differences of bio-ADM levels, outcomes (septic shock, 90-and 180-day mortality) did not differ significantly depending on the focus of infection (Fig. 6). A slight trend towards higher 180-day mortality in urinary tract infections was not significant (p = 0.2).

## Discussion

This post-hoc-analysis of the HYPRESS trial evaluated bio-ADM at different cut-offs as predictors of septic shock, 90-day and 180-day mortality as well as the potential of bio-ADM as a marker to identify subgroups of patients that might benefit from hydrocortisone treatment. To our knowledge, this is the first study investigating the biological interaction of steroids and bio-ADM in sepsis patients.

As reported in previous studies [28–30], elevated levels of bio-ADM on admission are associated with sepsis severity, vasopressor requirement and mortality. We could confirm a correlation of bio-ADM values on admission and onset of shock within 14 days as well as with 90- and 180-day mortality. Consistently, elevated bio-ADM levels at baseline were associated with higher disease severity, as expressed in higher SOFA and APACHE scores, higher incidence of organ (in particular renal) dysfunction, and with higher levels of markers of infection and inflammation (PCT and CRP). The highly differing bio-ADM cut-offs for prediction of outcomes renders clinical interpretation difficult, based on the current knowledge. The low cut-off for prediction of septic shock (37 pg/mL bio-ADM) are largely in line with previous studies, e.g. levels > 43 pg/mL for COVID-19 30-day mortality [41], bio-ADM > 45 pg/mL for 30-day mortality in the total ICU population or bio-ADM > 37 pg/mL for identification of sepsis [42].

These studies are difficult to compare due to differing disease severity across the cohorts and bio-ADM levels correlating with disease severity (initially shown by [43]). Similar results in several subsequent studies and our data corroborate this finding. We therefore conclude, that even slightly elevated levels of bio-ADM should attract special attention and thus, bio-ADM may be a useful additional parameter in the prognostic assessment of sepsis patients for early risk stratification.

Bio-ADM levels > 70 pg/mL have been used to predict sepsis mortality, e.g. in the AdrenOSS1 trial [28]. Of note, a level of 73.1 pg/mL was the best cut-off for prediction of 180-day mortality in our study (Table 3). For bio-ADM-guided interventions, the use of higher cut-off levels seems plausible in order to limit the risk of misidentifying patients. In the AdrenOSS-2 biomarker guided trial, bio-ADM levels > 70 pg/mL have been evaluated for safety and tolerability of the ADM antibody adrecizumab in septic shock patients [44]. While adrecizumab was well tolerated, its stabilisation of bio-ADM levels did not improve clinical outcomes in patients with septic shock [44].

Although retrospectively, we followed a similar approach to evaluate, if indirectly targeting bio-ADM levels by HC treatment could improve sepsis outcomes. However, bio-ADM levels were not associated with HC treatment response as none of the outcomes investigated differed between HC- and placebo group when stratified by bio-ADM levels.

Recent discussions focus on the so far neglected impact of focus of infection and systemic response biomarkers that might reflect local host responses inadequately [45]. We found clear differences in bio-ADM levels of patients with pneumonia and urogenital infections, the latter being associated with significantly higher bio-ADM levels. Mortality in urogenital sepsis is usually low compared to pulmonary sepsis [46–50]. Surprisingly, in our study, main patient outcomes did not differ depending on the focus of infection, irrespective of distinctive bio-ADM levels and renal dysfunction contributing strongest to higher SOFA scores in high bio-ADM subgroups. Our findings are, however, consistent with an association of renal dysfunction and disease severity with plasma ADM levels reported previously [22]. As the kidneys are commonly affected in sepsis [51] and adrenomedullin is also produced in the kidneys, in particular in hypoxic conditions [52], this might be an early sign of renal damage and a possible host response to counteract renal tissue damage by increasing renal blood flow and thus diuresis.

Regarding the still poorly understood compartmentalisation of host responses, a closer investigation of these correlations would be intriguing. Unfortunately, sample numbers in the HYPRESS cohort are not sufficient to perform detailed statistical analysis on respective subgroups, thus forbidding any conjectures regarding treatment-related effects. However, we consider further investigations of differing biomarker levels and possibly treatment effects depending on the source of infection of outstanding interest.

Differences in patient outcome may depend on the study design and patient characteristics. As recently demonstrated, sex of patients, as well as type, timing, and duration of corticosteroid treatment might affect patient outcome [15]. In this post-hoc analysis, the analysed subgroups differed in their patient characteristics, including sex, renal dysfunction, disease severity, and focus of infection, but not influencing the primary endpoint (septic shock).

So far, no gender-specific differences in bio-ADM have been reported in literature. As our data confirms the association of increased bio-ADM levels with renal dysfunction and thus its potential as an early marker of renal damage, which might render bio-ADM a useful tool in clinical decision making, e.g. the early initiation of renal protective strategies.

The highly variable responses of critically ill patients to corticosteroids can be sign of differences in activated signalling pathways or disease stages. For instance, a recent study showed that certain immunocompetent phenotypes might be associated with high mortality when treated with hydrocortisone, as opposed to those with an immune suppressed phenotype [15]. Briegel and colleagues reported different functional steroid reserves in critically ill sepsis patients by evaluating steroid profiles in combination with a corticotropin stimulation test providing information about steroidogenesis and for the prediction of shock development and mortality [53]. They showed a worse clinical outcome when the mineralocorticoid pathway was less responsive compared to the glucocorticoid pathway in the corticotropin test. Given that septic patients display a wide variation of molecular signatures [6, 53–56], innovative biomarkers could be helpful in identifying patients who may benefit from hydrocortisone therapy, while preventing harm to others.

Whereas our study corroborates the previously documented association of bio-ADM levels with disease severity and potential value for bio-ADM in prognosis, predictive enrichment of patient subgroups benefitting from HC treatment failed based on our data. Nonetheless, pursuing the search for biomarkers indicative of HC benefit or harm addresses a major medical need. To optimize existing low-cost and readily available treatments is of outstanding relevance, in particular for the use in low resource settings.

### Limitations of the study

Some limitations of our study have to be addressed. Analyses were performed post hoc, and results should therefore be regarded as hypothesis-generating. The strongly differing cut-offs for distinct outcome measures limit clinical interpretation and utility. Due to the size of the cohort, the current analyses may lack of powers and did not allow further investigation of the striking differences depending on focus of infection. Our findings may not be extrapolated to sicker patients as septic shock was an exclusion criterion. The mortality reported in this post-hoc analysis was comparatively low and comparable to the mortality reported in recent CAP studies [57].

## Conclusion

This study shows that levels of bio-ADM of 37 pg/mL or more are associated with increased incidence of shock and increased mortality in adult sepsis patients.We found no evidence that the response to hydrocortisone therapy can be modified or predicted by bio-ADM levels.

## Supplementary Information

Below is the link to the electronic supplementary material.

Supplementary file1 (DOCX 438 KB)

## Data Availability

No datasets were generated or analysed during the current study.

## Abbreviations

APACHE Score: Acute Physiology and Chronic Health Evaluation Score
Bio-ADM: Bioactive adrenomedullin
HC: Hydrocortisone
ICU: Intensive Care Unit
ITT population: Intention-to-treat- population
PP population: Per protocol population
SAPS Score: Simplified Acute Physiology Score
SIRS: Systemic inflammatory response syndrome
SOFA: Sepsis-related Organ Failure Assessment
