# Supplementary material for: Clinical value of circulating bioactive adrenomedullin for prediction of outcome and hydrocortisone response in sepsis patients—a post-hoc analysis of the HYPRESS trial
Source: Infection. 2025 May 30;53(6):2383–95. doi: 10.1007/s15010-025-02569-x (PMC12675558; doi:10.1007/s15010-025-02569-x)
Supplement: Supplementary file 1 — Supplementary file1 (DOCX 438 KB) [file 15010_2025_2569_MOESM1_ESM.docx]

Supplemental digital content to

**Clinical value of circulating bioactive adrenomedullin for prediction of outcome and hydrocortisone response in sepsis patients – a post-hoc analysis of the HYPRESS trial**

Caroline Neumann¹, Margit Leitner¹, Frank Bloos¹, Dorothea Lange^2^, Holger Bogatsch^3^, Djillali Annane^4^, Jerôme Fleuriet^4^, Josef Briegel^2#^, Michael Bauer¹^#^

and the SepNet Critical Care Trials Group and iRECORDS collaborators

#shared authorships

¹Department of Anaesthesiology and Intensive Care Medicine, Jena University Hospital, Am Klinikum 1, 07747 Jena, Germany

^2^Department of Anaesthesiology, LMU University Hospital, LMU Munich, Marchioninistraße 15, 81377 Munich, Germany

^3^Institute for Medical Informatics, Statistics and Epidemiology (IMISE) and Clinical Trial Centre, Leipzig University, Härtelstraße 16-18, 04107 Leipzig, Germany

^4^ IHU PROMETHEUS, Comprehensive Sepsis Centre, Hôpital Raymond Poincaré, Assistance Publique - Hôpitaux de Paris (APHP), 104 Boulevard Raymond Poincaré, 92380 Garches, France

Corresponding author:

Dr. med. Caroline Neumann, DESA, EDIC, Infectious Diseases Specialist
Department of Anaesthesiology and Intensive Care Medicine,
Jena University Hospital,
Am Klinikum 1, 07747 Jena, Germany
E-Mail: caroline.neumann@med.uni-jena.de

**Suppl. Table 1:** Baseline characteristics of the study population by treatment arm.

| **Characteristic** | **N** | **Overall**, N = 317*^1^* | **Placebo**, N = 153*^1^* | **HC**, N = 164*^1^* | **p-value***^2^* |
| --- | --- | --- | --- | --- | --- |
| Age (years) | 317 | 68.0 (55.0-75.0) | 68.0 (56.0-74.0) | 67.5 (55.0-76.0) | 0.7 |
| Male sex | 317 | 205 / 317 (65%) | 96 / 153 (63%) | 109 / 164 (66%) | 0.5 |
| Body Mass Index | 316 | 26.1 (23.4-29.7) | 26.3 (23.4-29.7) | 25.3 (23.3-29.7) | 0.4 |
| SOFA score | 267 | 6.0 (5.0-8.0) | 6.0 (5.0-8.0) | 6.0 (5.0-8.0) | 0.5 |
| Sub-SOFA respiratory system | 299 | 3.0 (2.0-3.0) | 3.0 (2.0-3.0) | 3.0 (2.0-3.0) | 0.6 |
| Sub-SOFA nervous system | 317 | 0.0 (0.0-1.0) | 0.0 (0.0-1.0) | 0.0 (0.0-1.0) | 0.7 |
| Sub-SOFA cardiovascular system | 310 | 1.0 (0.0-1.0) | 1.0 (0.0-1.0) | 1.0 (0.0-1.0) | 0.4 |
| Sub-SOFA liver | 290 | 0.0 (0.0-1.0) | 0.0 (0.0-0.0) | 0.0 (0.0-1.0) | 0.5 |
| Sub-SOFA coagulation | 316 | 0.0 (0.0-1.0) | 0.0 (0.0-1.0) | 0.0 (0.0-1.0) | 0.07 |
| Sub-SOFA kidneys | 315 | 1.0 (0.0-2.0) | 1.0 (0.0-2.0) | 1.0 (0.0-2.0) | 0.2 |
| APACHE score | 317 | 18.0 (15.0-22.0) | 18.0 (15.0-22.0) | 18.0 (14.0-22.0) | 0.6 |
| **SIRS criteria** |  |  |  |  |  |
| Temperature ≤36°C or ≥38°C | 317 | 240 / 317 (76%) | 121 / 153 (79%) | 119 / 164 (73%) | 0.2 |
| Heart rate ≥90 beats/min | 317 | 292 / 317 (92%) | 146 / 153 (95%) | 146 / 164 (89%) | **0.035** |
| Tachypnea, hypocapnia, or mechanical ventilation | 316 | 281 / 316 (89%) | 140 / 153 (92%) | 141 / 163 (87%) | 0.2 |
| Leukocytosis, leukopenia, or left shift | 317 | 233 / 317 (74%) | 112 / 153 (73%) | 121 / 164 (74%) | >0.9 |
| **Physiological variables^*^** |  |  |  |  |  |
| Max. heart rate (1/min) | 316 | 115.0 (101.0-129.0) | 115.0 (101.0-129.0) | 115.0 (101.0-128.5) | 0.7 |
| Min. MAP (mmHg) | 299 | 65.0 (60.0-75.0) | 65.0 (60.0-75.0) | 65.0 (59.0-74.3) | 0.7 |
| Max. respiratory rate (1/min) | 270 | 29.0 (24.0-33.0) | 29.0 (24.0-33.0) | 29.0 (23.8-33.0) | 0.8 |
| Urinary excretion (ml/24h) | 307 | 1,810.0 (1,059.3-3,016.0) | 1,932.5 (1,063.0-3,207.5) | 1,800.0 (1,058.5-2,785.0) | 0.2 |
| Min. PaO_2_/FiO_2_ ratio (mmHg) | 289 | 188.0 (130.0-254.0) | 181.8 (129.9-239.8) | 192.0 (130.0-267.8) | 0.3 |
| **Organ dysfunction** |  |  |  |  |  |
| Acute encephalopathy^1^ | 315 | 77 / 315 (24%) | 39 / 153 (25%) | 38 / 162 (23%) | 0.7 |
| Renal dysfunction² | 317 | 124 / 317 (39%) | 64 / 153 (42%) | 60 / 164 (37%) | 0.3 |
| Coagulopathy^3^ | 317 | 56 / 317 (18%) | 23 / 153 (15%) | 33 / 164 (20%) | 0.2 |
| Arterial hypoxemia^4^ | 316 | 214 / 316 (68%) | 104 / 152 (68%) | 110 / 164 (67%) | 0.8 |
| Microcirculatory dysfunction^5^ | 316 | 105 / 316 (33%) | 48 / 153 (31%) | 57 / 163 (35%) | 0.5 |
| **Laboratory values** |  |  |  |  |  |
| Leukocytes min. [G/l] | 314 | 12.0 (7.9-18.0) | 12.0 (7.9-18.0) | 12.0 (7.9-17.2) | >0.9 |
| Platelets, min. [G/l] | 316 | 196.5 (132.8-280.5) | 198.0 (141.0-284.0) | 195.0 (129.5-275.5) | 0.5 |
| Max. bilirubin (µmol/l) | 259 | 12.0 (8.0-21.0) | 14.0 (8.0-20.5) | 12.0 (7.0-21.0) | 0.9 |
| Max. serum creatinine (µmol/l) | 249 | 115.0 (71.0-206.0) | 128.0 (87.0-220.0) | 106.0 (68.0-189.5) | **0.025** |
| Max. urea (mmol/l) | 303 | 9.5 (6.0-15.0) | 10.0 (6.0-16.3) | 9.0 (5.5-13.8) | 0.3 |
| CRP level [mg/l] | 240 | 219.0 (138.8-293.0) | 226.0 (143.8-288.0) | 211.0 (134.8-292.9) | 0.8 |
| PCT level [ng/ml] | 227 | 3.0 (1.0-10.0) | 3.0 (1.0-8.0) | 3.0 (1.0-13.0) | 0.3 |
| Max. lactate at baseline [mmol/l] | 307 | 1.8 (1.2-2.6) | 1.6 (1.2-2.7) | 1.8 (1.3-2.6) | 0.2 |
| bio-ADM [pg/mL] | 317 | 55.6 (33.4-88.1) | 56.8 (33.4-86.4) | 52.8 (33.6-93.1) | >0.9 |
| **Focus of infection** | 317 |  |  |  |  |
| Pneumonia |  | 122 / 317 (38%) | 69 / 153 (45%) | 53 / 164 (32%) | **0.026** |
| Other infections of upper or lower airways |  | 17 / 317 (5.4%) | 9 / 153 (5.9%) | 8 / 164 (4.9%) | 0.8 |
| Thoracic (Empyem / Mediastinitis) |  | 8 / 317 (2.5%) | 1 / 153 (0.7%) | 7 / 164 (4.3%) | 0.13 |
| Gastrointestinal |  | 14 / 317 (4.4%) | 5 / 153 (3.3%) | 9 / 164 (5.5%) | 0.6 |
| Primary bacteremia |  | 4 / 317 (1.3%) | 2 / 153 (1.3%) | 2 / 164 (1.2%) | >0.9 |
| Catheter-associated infection |  | 8 / 317 (2.5%) | 2 / 153 (1.3%) | 6 / 164 (3.7%) | 0.4 |
| Bones/soft tissues |  | 26 / 317 (8.2%) | 11 / 153 (7.2%) | 15 / 164 (9.1%) | 0.8 |
| Surgical wound infection |  | 6 / 317 (1.9%) | 3 / 153 (2.0%) | 3 / 164 (1.8%) | >0.9 |
| Intra-abdominal |  | 59 / 317 (19%) | 24 / 153 (16%) | 35 / 164 (21%) | 0.4 |
| Central nervous system |  | 3 / 317 (0.9%) | 1 / 153 (0.7%) | 2 / 164 (1.2%) | 0.9 |
| Genitourinary |  | 38 / 317 (12%) | 17 / 153 (11%) | 21 / 164 (13%) | 0.8 |
| Unknown source of infection |  | 53 / 317 (17%) | 27 / 153 (18%) | 26 / 164 (16%) | 0.7 |
| Acute nosocomial infection | 317 | 68 / 317 (21%) | 30 / 153 (20%) | 38 / 164 (23%) | 0.4 |

*^1^*Median (25%-75%); n / N (%)

*^2^*Wilcoxon rank sum test; Pearson's Chi-squared test; Fisher's exact test
Abbrev.: bio-ADM: bioactive adrenomedullin; SOFA: Sepsis-related Organ Failure Assessment; APACHE Score: Acute Physiology And Chronic Health Evaluation Score; SAPS Score: Simplified Acute Physiology Score; SIRS: Systemic Inflammatory Response Syndrome; HC: hydrocortisone.
*24 h time interval before study inclusion; ¹ reduced vigilance, restlessness, disorientation, delirium, unaffected by psychotropic drugs; ² oliguria: < 0.5 mL/kg/h for at least 2 hours despite adequate volume replacement and/or creatinine increase ≥ 2 times above the reference value of the respective laboratory and/or renal replacement therapy; ³ thrombocytopenia ≤ 100,000/µL or more than a 30% decrease from baseline within 24 hours (not caused by bleeding or immunological factors); ⁴ PaO2 ≤ 75 mmHg [≤ 10 kPa] under room air or a PaO2/FiO2 ≤ 250 mmHg [≤ 33 kPa] under oxygen administration. (Not due to pre-existing heart or lung disease); ⁵ Lactate > 1.5 times the upper reference range and/or base deficit ≥ 5 mmol/L and/or metabolic acidosis with pH < 7.3 and/or impaired capillary reperfusion and/or marbling and/or significant edema in capillary leak syndrome.

**Suppl. Table 2:** Main outcomes of the study population by treatment arm.

| **Outcomes** | **N** | **Overall**, N = 317*^1^* | **Placebo**, N = 153*^1^* | **HC**, N = 164*^1^* | **p-value***^2^* |
| --- | --- | --- | --- | --- | --- |
| Septic shock within 14 days | 317 | 64 / 317 (20%) | 30 / 153 (20%) | 34 / 164 (21%) | 0.8 |
| 28-day survival | 302 | 289 / 302 (96%) | 142 / 147 (97%) | 147 / 155 (95%) | 0.5 |
| 90-day survival | 284 | 256 / 284 (90%) | 126 / 140 (90%) | 130 / 144 (90%) | >0.9 |
| 180-day survival | 308 | 233 / 308 (76%) | 117 / 149 (79%) | 116 / 159 (73%) | 0.3 |
| ICU length of stay [d] | 315 | 7.0 (4.5-15.0) | 7.0 (5.0-16.0) | 7.0 (4.0-13.0) | 0.3 |
| Hospital length of stay [d] | 315 | 22.0 (14.0-44.0) | 22.0 (13.0-39.0) | 23.5 (15.0-47.0) | 0.2 |
| Days with mechanical ventilation until day 28 (on ICU) | 315 | 1.0 (0.0-6.0) | 2.0 (0.0-7.0) | 1.0 (0.0-5.0) | 0.3 |
| Δ SOFA score | 246 | 1.0 (-1.0-3.0) | 1.0 (-1.0-3.0) | 1.0 (-1.0-2.3) | 0.9 |
| Days with RRT until day 28 (on ICU)*^3^* | 315 | 0.7 (2.7) | 0.7 (2.8) | 0.8 (2.7) | >0.9 |
| Mean SOFA score until day 14 | 276 | 5.0 (4.0-7.0) | 5.0 (4.0-7.0) | 5.0 (4.0-6.0) | 0.7 |

*^1^*Median (25%-75%); n / N (%)

*^2^*Wilcoxon rank sum test; Pearson's Chi-squared test
*^3^*Mean (sd); Median and interquartile range are 0.

| 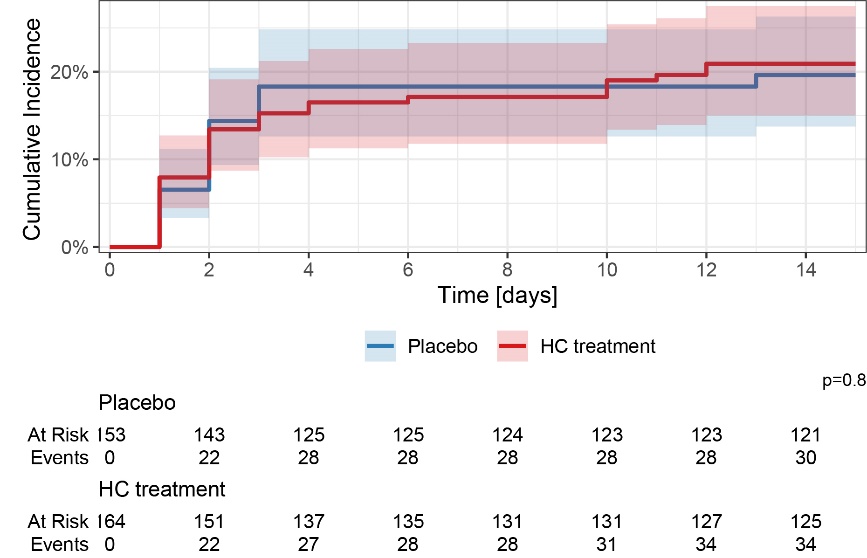 | **Suppl. Figure 1:** Cumulative incidence of septic shock within 14 days by treatment group. |
| --- | --- |

| 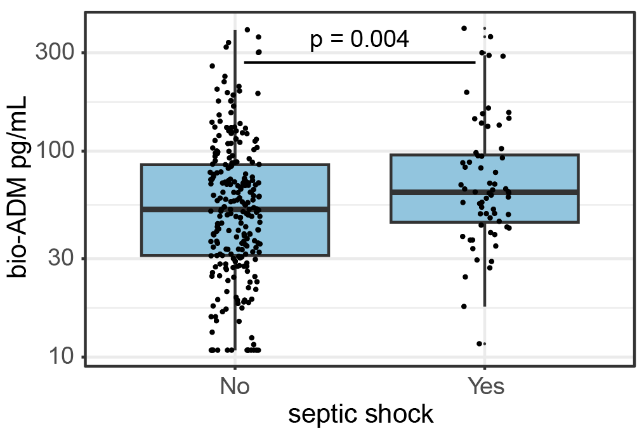 | **Suppl. Figure 2** Baseline bio-ADM levels of patients with or without development of shock within 14 days. *P*-value as determined by two sample t-test on log-transformed bio-ADM values. |
| --- | --- |

| 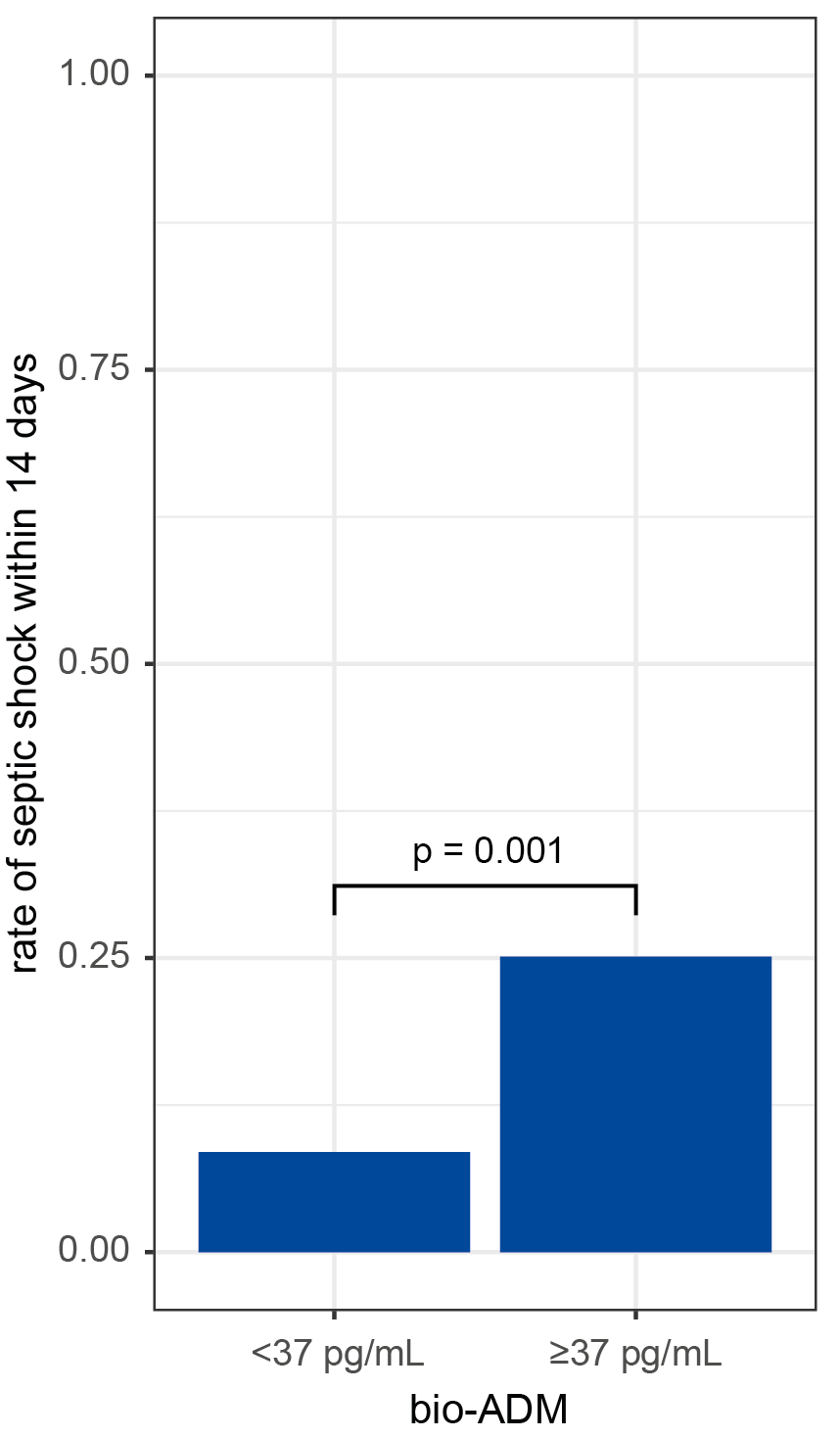 | **Suppl. Figure 3** Septic shock within 14 days in patients with baseline bio-ADM levels below 37 pg/mL or ≥37 pg/mL. *P*-values based on Pearson's Chi-squared test with Yates' continuity correction. |
| --- | --- |

**Suppl. Table 3:** Logistic regression of bio-ADM subgroups (below or above 37 pg/mL) and interaction with treatment arms for the primary outcome, **septic shock within 14 days**.

| **Characteristic** |  | **OR** | **95% CI** | **p-value** |
| --- | --- | --- | --- | --- |
| bio-ADM group | <37 pg/mL | — | — |  |
|  | ≥37 pg/mL | 4.67 | 1.53, 20.3 | **0.016** |
| Intervention | Placebo | — | — |  |
|  | Hydrocortisone | 1.59 | 0.37, 8.15 | 0.54 |
| Interaction | bio-ADM ≥ 37 pg/mL * HC | 0.64 | 0.11, 3.10 | 0.58 |

Abbreviations: OR = Odds Ratio, CI = Confidence Interval, HC = hydrocortisone, bio-ADM = bioactive adrenomedullin

**Suppl. Table 4:** Adjusted logistic regression (including age, sex and renal dysfunction) of bio-ADM subgroups (below or above 37 pg/mL) and interaction with treatment arms for the primary outcome, **septic shock within 14 days**.

| **Characteristic** |  | **OR** | **95% CI** | **p-value** |
| --- | --- | --- | --- | --- |
| bio-ADM group | <37 pg/mL | — | — |  |
|  | ≥37 pg/mL | 4.40 | 1.41, 19.5 | **0.022** |
| Intervention | Placebo | — | — |  |
|  | Hydrocortisone | 1.59 | 0.37, 8.20 | 0.54 |
| Sex | Female | — | — |  |
|  | Male | 1.56 | 0.86, 2.92 | 0.15 |
| Age |  | 1.00 | 0.99, 1.03 | 0.66 |
| Renal dysfunction^1^ | No | — | — |  |
|  | Yes | 1.34 | 0.75, 2.39 | 0.32 |
| Interaction | bio-ADM ≥ 37 pg/mL * HC | 0.63 | 0.11, 3.11 | 0.58 |

Abbreviations: OR = Odds Ratio, CI = Confidence Interval, HC = hydrocortisone, bio-ADM = bioactive adrenomedullin

^1^ Renal dysfunction present, if fulfilling the criteria: oliguria: < 0.5 mL/kg/h for at least 2 hours despite adequate volume replacement and/or creatinine increase ≥ 2 times above the reference value of the respective laboratory and/or renal replacement therapy

| **A**  **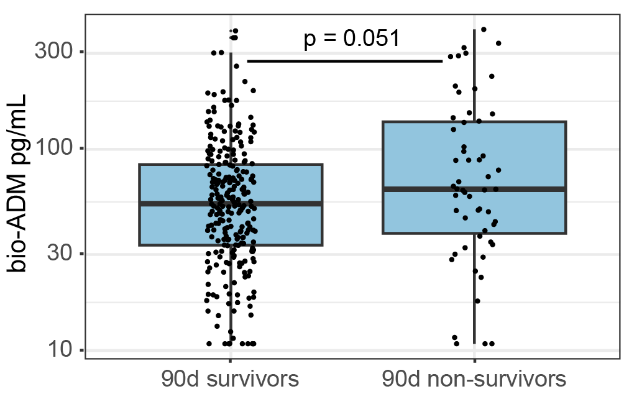** | **B**  **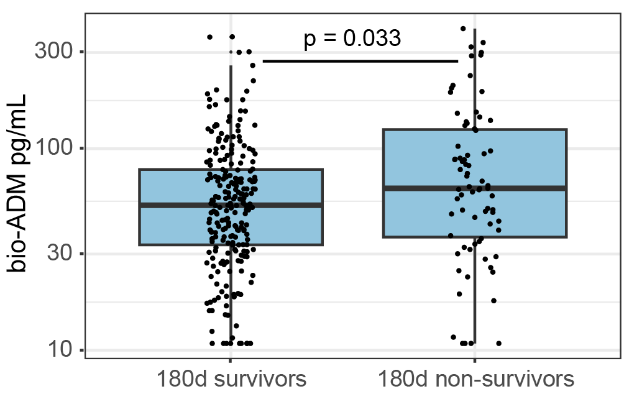** |
| --- | --- |
| **Suppl. Figure 4:** Baseline bio-ADM levels of patients **(A)** by 90-day survival and **(B)** by 180-day survival. P-value as determined by two sample t-test on log-transformed bio-ADM values. | |

| 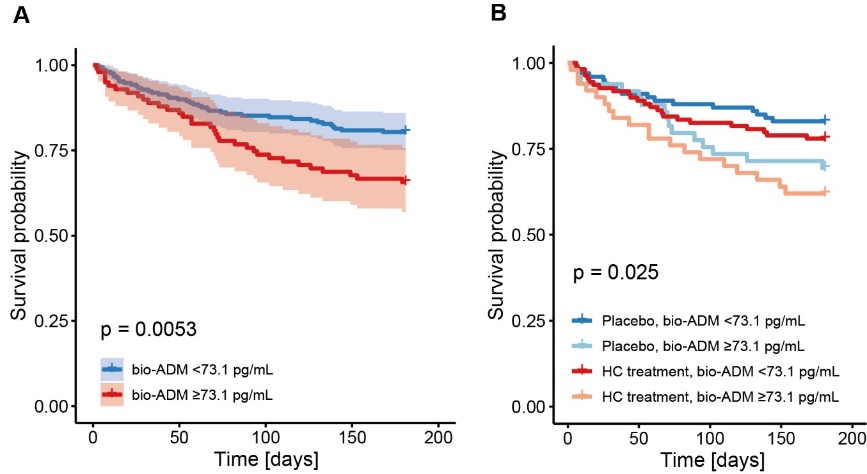 | **Suppl. Figure 5:** 180-day mortality **(A)** by bio-ADM groups <73.1 and ≥73.1 pg/mL and **(B)** by bio-ADM groups <73.1 and ≥73.1 pg/mL and HC treatment. |
| --- | --- |

**Suppl. Table 5:** Baseline characteristics of the patient population by bio-ADM at cut-off **73.1 pg/mL** and treatment arm.

| **Characteristic** | **N** | **Overall**, N = 317*^1^* | **<73.1 pg/mL Placebo**, N = 104*^1^* | **<73.1 pg/mL HC**, N = 112*^1^* | **over 73.1 pg/mL Placebo**, N = 49*^1^* | **over 73.1 pg/mL trial**, N = 52*^1^* | **p-value***^2^* |
| --- | --- | --- | --- | --- | --- | --- | --- |
| Age (years) | 317 | 68.0 (55.0-75.0) | 68.0 (55.8-74.0) | 66.5 (54.8-75.3) | 68.0 (57.0-76.0) | 71.0 (60.5-76.3) | 0.8 |
| Male sex | 317 | 205 / 317 (65%) | 73 / 104 (70%) | 77 / 112 (69%) | 23 / 49 (47%) | 32 / 52 (62%) | **0.027** |
| Body Mass Index | 316 | 26.1 (23.4-29.7) | 26.3 (23.4-29.4) | 24.6 (23.2-29.3) | 26.9 (23.9-31.1) | 26.7 (23.8-32.9) | 0.14 |
| SOFA score | 267 | 6.0 (5.0-8.0) | 5.0 (4.0-7.0) | 6.0 (4.0-7.0) | 7.0 (5.0-8.0) | 7.0 (5.0-9.0) | **<0.001** |
| Sub-SOFA respiratory system | 299 | 3.0 (2.0-3.0) | 3.0 (2.0-3.0) | 3.0 (2.0-3.0) | 3.0 (2.0-3.0) | 2.0 (2.0-3.0) | **0.002** |
| Sub-SOFA nervous system | 317 | 0.0 (0.0-1.0) | 0.0 (0.0-1.0) | 0.0 (0.0-1.0) | 0.0 (0.0-1.0) | 0.0 (0.0-1.0) | 0.4 |
| Sub-SOFA cardiovascular system | 310 | 1.0 (0.0-1.0) | 1.0 (0.0-1.0) | 1.0 (0.0-1.0) | 1.0 (0.0-1.0) | 1.0 (1.0-1.0) | 0.3 |
| Sub-SOFA liver | 290 | 0.0 (0.0-1.0) | 0.0 (0.0-0.25) | 0.0 (0.0-0.0) | 0.0 (0.0-0.0) | 0.0 (0.0-1.0) | 0.07 |
| Sub-SOFA coagulation | 316 | 0.0 (0.0-1.0) | 0.0 (0.0-1.0) | 0.0 (0.0-1.0) | 0.0 (0.0-1.0) | 1.0 (0.0-2.0) | **0.002** |
| Sub-SOFA kidneys | 315 | 1.0 (0.0-2.0) | 0.0 (0.0-2.0) | 0.0 (0.0-1.0) | 2.0 (1.0-3.0) | 2.0 (1.0-3.25) | **<0.001** |
| APACHE score | 317 | 18.0 (15.0-22.0) | 17.5 (15.0-21.0) | 17.0 (14.0-21.0) | 19.0 (17.0-24.0) | 20.0 (16.0-24.0) | **0.026** |
| **SIRS criteria** |  |  |  |  |  |  |  |
| Temperature ≤36°C or ≥38°C | 317 | 240 / 317 (76%) | 88 / 104 (85%) | 86 / 112 (77%) | 33 / 49 (67%) | 33 / 52 (63%) | **0.014** |
| Heart rate ≥90 beats/min | 317 | 292 / 317 (92%) | 100 / 104 (96%) | 105 / 112 (94%) | 46 / 49 (94%) | 41 / 52 (79%) | **0.004** |
| Tachypnea, hypocapnia, or mechanical ventilation | 316 | 281 / 316 (89%) | 97 / 104 (93%) | 97 / 112 (87%) | 43 / 49 (88%) | 44 / 51 (86%) | 0.4 |
| Leukocytosis, leukopenia, or left shift |  | 233 / 317 (74%) | 76 / 104 (73%) | 80 / 112 (71%) | 36 / 49 (73%) | 41 / 52 (79%) | 0.8 |
| **Physiological variables *** |  |  |  |  |  |  |  |
| Max. heart rate (1/min) | 316 | 115.0 (101.0-129.0) | 112.5 (101.8-129.3) | 117.0 (103.0-128.5) | 120.0 (100.0-128.0) | 110.0 (94.0-127.8) | 0.5 |
| Min. MAP (mmHg) | 299 | 65.0 (60.0-75.0) | 65.0 (60.0-76.0) | 65.0 (60.0-78.0) | 64.0 (58.3-71.8) | 63.0 (57.3-68.0) | 0.11 |
| Max. respiratory rate (1/min) | 270 | 29.0 (24.0-33.0) | 29.0 (24.0-34.0) | 30.0 (24.3-34.0) | 27.0 (24.0-32.0) | 25.5 (22.0-31.8) | 0.093 |
| Urinary excretion (ml/24h) | 307 | 1,810.0 (1,059.3-3,016.0) | 2,260.0 (1,360.0-3,390.0) | 2,000.0 (1,308.9-3,090.0) | 1,418.0 (906.5-2,790.0) | 1,091.0 (485.0-2,057.1) | **<0.001** |
| Min. PaO_2_/FiO_2_ ratio (mmHg) | 289 | 188.0 (130.0-254.0) | 179.0 (132.5-240.5) | 176.5 (115.3-231.5) | 185.0 (123.0-237.0) | 242.0 (190.0-295.0) | **<0.001** |
| **Organ dysfunction** |  |  |  |  |  |  |  |
| Acute encephalopathy^1^ | 315 | 77 / 315 (24%) | 23 / 104 (22%) | 24 / 110 (22%) | 16 / 49 (33%) | 14 / 52 (27%) | 0.4 |
| Renal dysfunction² | 317 | 124 / 317 (39%) | 38 / 104 (37%) | 25 / 112 (22%) | 26 / 49 (53%) | 35 / 52 (67%) | **<0.001** |
| Coagulopathy^3^ | 317 | 56 / 317 (18%) | 12 / 104 (12%) | 21 / 112 (19%) | 11 / 49 (22%) | 12 / 52 (23%) | 0.2 |
| Arterial hypoxemia^4^ | 316 | 214 / 316 (68%) | 75 / 103 (73%) | 82 / 112 (73%) | 29 / 49 (59%) | 28 / 52 (54%) | **0.030** |
| Microcirculatory dysfunction^5^ | 316 | 105 / 316 (33%) | 25 / 104 (24%) | 32 / 112 (29%) | 23 / 49 (47%) | 25 / 51 (49%) | **0.002** |
| **Laboratory values** |  |  |  |  |  |  |  |
| Leukocytes max. [G/l] | 256 | 14.8 (10.0-21.0) | 15.9 (10.0-21.0) | 14.8 (11.0-20.1) | 15.0 (9.0-21.0) | 12.9 (8.8-23.0) | 0.9 |
| Platelets, min. [G/l] | 316 | 196.5 (132.8-280.5) | 201.0 (146.8-281.0) | 216.0 (138.0-300.0) | 196.0 (133.0-298.0) | 142.5 (92.5-224.5) | **0.013** |
| Max. bilirubin (µmol/l) | 259 | 12.0 (8.0-21.0) | 14.0 (8.0-20.8) | 12.0 (8.0-19.5) | 11.0 (8.0-19.0) | 14.0 (7.0-32.0) | 0.6 |
| Max. serum creatinine (µmol/l) | 249 | 115.0 (71.0-206.0) | 115.0 (70.8-184.5) | 88.0 (62.0-124.0) | 177.0 (116.5-308.5) | 194.0 (103.0-294.5) | **<0.001** |
| Max. urea (mmol/l) | 303 | 9.5 (6.0-15.0) | 8.0 (5.0-13.0) | 7.1 (5.0-11.0) | 14.0 (10.0-20.8) | 13.0 (7.7-20.0) | **<0.001** |
| CRP level [mg/l] | 240 | 219.0 (138.8-293.0) | 218.0 (144.5-262.0) | 176.0 (126.0-269.0) | 243.0 (133.5-331.5) | 258.0 (183.0-318.0) | 0.094 |
| PCT level [ng/ml] | 227 | 3.0 (1.0-10.0) | 1.0 (0.5-4.0) | 1.5 (1.0-6.0) | 4.0 (2.0-13.0) | 11.0 (5.0-34.5) | **<0.001** |
| Max. lactate at baseline [mmol/l] | 307 | 1.8 (1.2-2.6) | 1.5 (1.1-2.3) | 1.8 (1.3-2.6) | 2.1 (1.4-3.2) | 2.0 (1.1-2.7) | **0.035** |
| bioADM [pg/mL] | 317 | 55.6 (33.4-88.1) | 41.0 (28.9-57.6) | 40.0 (27.5-55.1) | 100.1 (87.7-144.2) | 126.8 (99.2-179.4) | **<0.001** |
| **Focus of infection** | 317 |  |  |  |  |  |  |
| Pneumonia |  | 122 / 317 (38%) | 51 / 104 (49%) | 46 / 112 (41%) | 18 / 49 (37%) | 7 / 52 (13%) | **<0.001** |
| Other infections of upper or lower airways |  | 17 / 317 (5.4%) | 8 / 104 (7.7%) | 8 / 112 (7.1%) | 1 / 49 (2.0%) | 0 / 52 (0%) | 0.15 |
| Thoracic (Empyem / Mediastinitis) |  | 8 / 317 (2.5%) | 1 / 104 (1.0%) | 6 / 112 (5.4%) | 0 / 49 (0%) | 1 / 52 (1.9%) | 0.3 |
| Gastrointestinal |  | 14 / 317 (4.4%) | 2 / 104 (1.9%) | 5 / 112 (4.5%) | 3 / 49 (6.1%) | 4 / 52 (7.7%) | 0.5 |
| Primary bacteremia |  | 4 / 317 (1.3%) | 0 / 104 (0%) | 1 / 112 (0.9%) | 2 / 49 (4.1%) | 1 / 52 (1.9%) | 0.3 |
| Catheter-associated infection |  | 8 / 317 (2.5%) | 1 / 104 (1.0%) | 3 / 112 (2.7%) | 1 / 49 (2.0%) | 3 / 52 (5.8%) | 0.5 |
| Bones/soft tissues |  | 26 / 317 (8.2%) | 8 / 104 (7.7%) | 8 / 112 (7.1%) | 3 / 49 (6.1%) | 7 / 52 (13%) | 0.6 |
| Surgical wound infection |  | 6 / 317 (1.9%) | 2 / 104 (1.9%) | 1 / 112 (0.9%) | 1 / 49 (2.0%) | 2 / 52 (3.8%) | 0.6 |
| Intra-abdominal |  | 59 / 317 (19%) | 13 / 104 (13%) | 22 / 112 (20%) | 11 / 49 (22%) | 13 / 52 (25%) | 0.4 |
| Central nervous system |  | 3 / 317 (0.9%) | 1 / 104 (1.0%) | 2 / 112 (1.8%) | 0 / 49 (0%) | 0 / 52 (0%) | 0.7 |
| Genitourinary |  | 38 / 317 (12%) | 7 / 104 (6.7%) | 5 / 112 (4.5%) | 10 / 49 (20%) | 16 / 52 (31%) | **<0.001** |
| Unknown source of infection |  | 53 / 317 (17%) | 21 / 104 (20%) | 20 / 112 (18%) | 6 / 49 (12%) | 6 / 52 (12%) | 0.4 |

*^1^* Median (25%-75%); n / N (%)
*^2^* Kruskal-Wallis rank sum test; Pearson's Chi-squared test; Fisher's exact test
Abbrev.: SOFA: Sequential Organ Failure Assessment; APACHE: Acute Physiology And Chronic Health Evaluation ; SAPS: simplified acute physiology score ; SIRS: systemic inflammatory response syndrome; MAP: mean arterial pressure ; PCT: procalcitonin; CRP: C-reactive protein; bio-ADM: bioactive adrenomedullin; ICU, intensive care unit. * 24 h time interval before study inclusion; ¹ reduced vigilance, restlessness, disorientation, delirium, unaffected by psychotropic drugs; ² oliguria: < 0.5 mL/kg/h for at least 2 hours despite adequate volume replacement and/or creatinine increase ≥ 2 times above the reference value of the respective laboratory and/or renal replacement therapy; ³ thrombocytopenia ≤ 100,000/µL or more than a 30% decrease from baseline within 24 hours (not caused by bleeding or immunological factors); ⁴ PaO2 ≤ 75 mmHg [≤ 10 kPa] under room air or a PaO2/FiO2 ≤ 250 mmHg [≤ 33 kPa] under oxygen administration. (Not due to pre-existing heart or lung disease); ⁵ Lactate > 1.5 times the upper reference range and/or base deficit ≥ 5 mmol/L and/or metabolic acidosis with pH < 7.3 and/or impaired capillary reperfusion and/or marbling and/or significant edema in capillary leak syndrome.

**Suppl. Table 6:** Main outcomes of the patient population by bio-ADM at cut-off **73.1 pg/mL** and treatment arm.

| **Outcomes** | **N** | **Overall**, N = 317*^1^* | **<73.1 cont**, N = 104*^1^* | **<73.1 trial**, N = 112*^1^* | **over 73.1 cont**, N = 49*^1^* | **over 73.1 trial**, N = 52*^1^* | **p-value***^2^* |
| --- | --- | --- | --- | --- | --- | --- | --- |
| 28-day survival | 302 | 289 / 302 (96%) | 96 / 100 (96%) | 102 / 107 (95%) | 46 / 47 (98%) | 45 / 48 (94%) | 0.8 |
| 90-day survival | 284 | 256 / 284 (90%) | 89 / 94 (95%) | 91 / 99 (92%) | 37 / 46 (80%) | 39 / 45 (87%) | 0.052 |
| 180-day survival | 308 | 233 / 308 (76%) | 83 / 100 (83%) | 85 / 109 (78%) | 34 / 49 (69%) | 31 / 50 (62%) | **0.025** |
| ICU length of stay [d] | 315 | 7.0 (4.5-15.0) | 7.0 (5.0-15.3) | 7.0 (4.0-12.8) | 8.0 (4.0-16.0) | 6.5 (4.0-21.8) | 0.8 |
| Hospital length of stay [d] | 315 | 22.0 (14.0-44.0) | 19.0 (12.0-33.3) | 21.5 (14.0-42.8) | 25.0 (16.0-57.0) | 29.0 (16.5-54.3) | **0.040** |
| Days with mech ventilation until D28 (on ICU) | 315 | 1.0 (0.0-6.0) | 2.0 (0.0-6.0) | 1.0 (0.0-5.0) | 2.0 (0.0-7.0) | 0.0 (0.0-5.0) | 0.5 |
| Δ SOFA score | 246 | 1.0 (-1.0-3.0) | 0.0 (-1.0-3.0) | 1.0 (-1.0-2.0) | 1.0 (-1.0-4.0) | 1.0 (-1.0-3.3) | 0.4 |
| Days with RRT until D28 (on ICU) | 315 | 0.7 (2.7) | 0.8 (3.1) | 0.6 (2.6) | 0.6 (2.0) | 1.2 (2.9) | 0.060 |

*^1^*Median (25%-75%); n / N (%)

*^2^*Wilcoxon rank sum test; Pearson's Chi-squared test
*^3^*Mean (sd); Median and interquartile range are 0.

| **A**  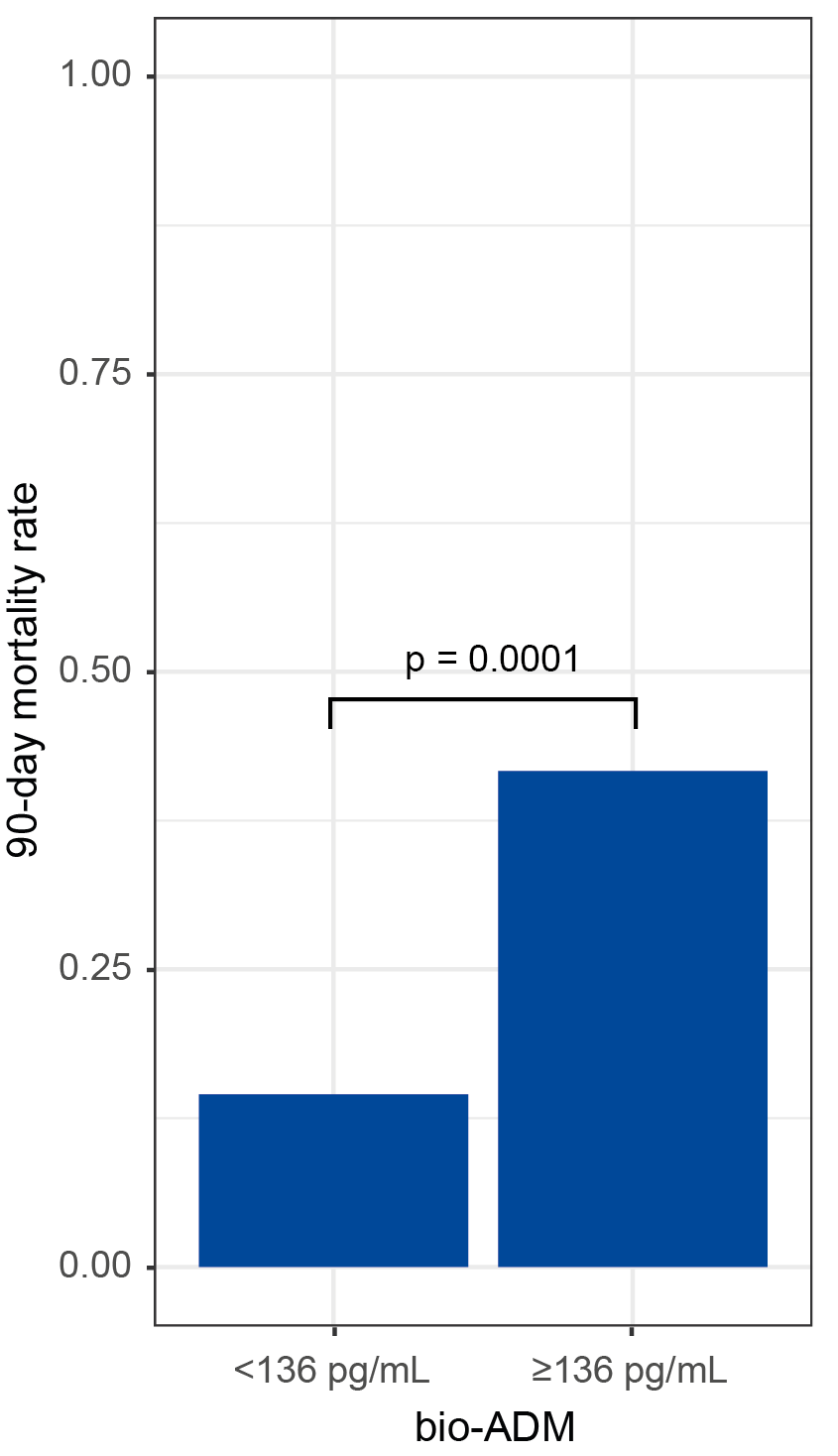 | **B**  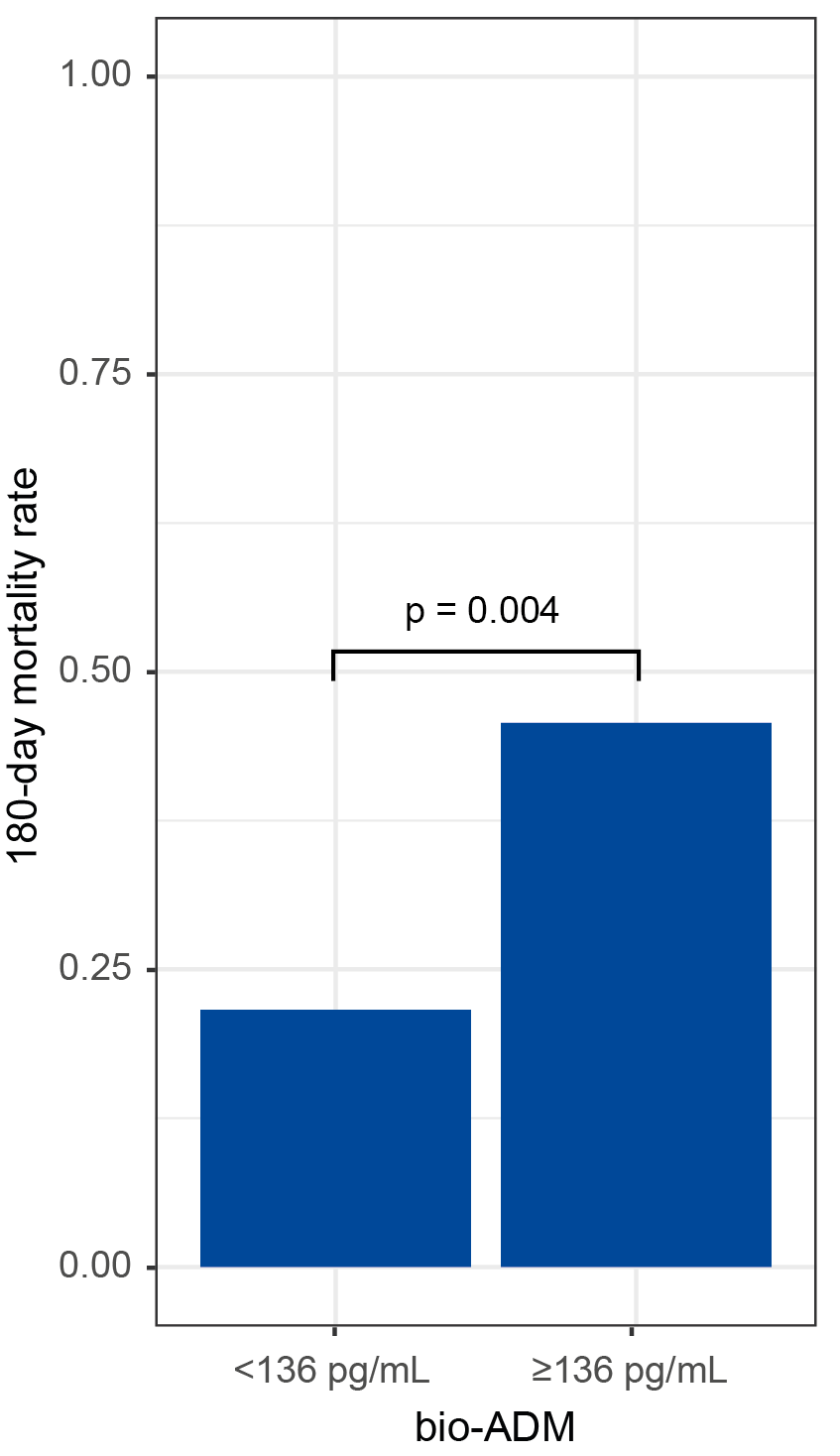 |
| --- | --- |
| **Suppl. Figure 6:** Mortality in defined bio-ADM subgroups. (A) 90-day mortality in patients with baseline bio-ADM levels below 136 pg/mL or ≥136 pg/mL. (B) 180-day mortality in patients with baseline bio-ADM levels below 136 pg/mL or ≥136 pg/mL. *P*-values based on Pearson's Chi-squared test with Yates' continuity correction. | |

**Suppl. Table 7:** Adjusted logistic regression (including age, sex and renal dysfunction) of bio-ADM subgroups (below or above 136 pg/mL) and interaction with treatment arms for **90-day mortality**.

| **Characteristic** |  | **OR** | **95% CI** | **p-value** |
| --- | --- | --- | --- | --- |
| bio-ADM group | <136 pg/mL | — | — |  |
|  | ≥136 pg/mL | 8.21 | 2.46, 27.9 | **<0.001** |
| Intervention | Placebo | — | — |  |
|  | Hydrocortisone | 1.53 | 0.77, 3.10 | 0.23 |
| Sex | Female | — | — |  |
|  | Male | 1.46 | 0.77, 2.90 | 0.26 |
| Age |  | 1.03 | 1.01, 1.06 | **0.012** |
| Renal dysfunction^1^ | No | — | — |  |
|  | Yes | 0.99 | 0.52, 1.87 | 0.98 |
| Interaction | bio-ADM ≥ 136 pg/mL * HC | 0.46 | 0.10, 2.09 | 0.31 |

Abbreviations: OR = Odds Ratio, CI = Confidence Interval, HC = hydrocortisone, bio-ADM = bioactive adrenomedullin

^1^ Renal dysfunction present, if fulfilling the criteria: oliguria: < 0.5 mL/kg/h for at least 2 hours despite adequate volume replacement and/or creatinine increase ≥ 2 times above the reference value of the respective laboratory and/or renal replacement therapy

**Suppl. Table 8:** Adjusted logistic regression (including age, sex and renal dysfunction) of bio-ADM subgroups (below or above 136 pg/mL) and interaction with treatment arms for **180-day mortality**.

| **Characteristic** |  | **OR** | **95% CI** | **p-value** |
| --- | --- | --- | --- | --- |
| bio-ADM group | <136 pg/mL | — | — |  |
|  | ≥136 pg/mL | 4.87 | 1.49, 16.0 | **0.008** |
| Intervention | Placebo | — | — |  |
|  | Hydrocortisone | 1.41 | 0.79, 2.55 | 0.25 |
| Sex | Female | — | — |  |
|  | Male | 1.39 | 0.78, 2.52 | 0.27 |
| Age |  | 1.04 | 1.02, 1.06 | **<0.001** |
| Renal dysfunction^1^ | No | — | — |  |
|  | Yes | 0.92 | 0.51, 1.61 | 0.76 |
| Interaction | bio-ADM ≥ 136 pg/mL * HC | 0.60 | 0.13, 2.99 | 0.51 |

Abbreviations: OR = Odds Ratio, CI = Confidence Interval, HC = hydrocortisone, bio-ADM = bioactive adrenomedullin

^1^ Renal dysfunction present, if fulfilling the criteria: oliguria: < 0.5 mL/kg/h for at least 2 hours despite adequate volume replacement and/or creatinine increase ≥ 2 times above the reference value of the respective laboratory and/or renal replacement therapy

**Suppl. Table 9:** Baseline statistics of the patient population by bioADM at cut-off **136 pg/mL** and treatment arm.

| **Characteristic** | **N** | **Overall**, N = 317*^1^* | **<136 Placebo**, N = 138*^1^* | **<136 HC**, N = 143*^1^* | **over 136 Placebo**, N = 15*^1^* | **over 136 HC**, N = 21*^1^* | **p-value***^2^* |
| --- | --- | --- | --- | --- | --- | --- | --- |
| Age (years) | 317 | 68.0 (55.0-75.0) | 68.0 (56.3-74.8) | 68.0 (55.5-76.0) | 68.0 (54.0-71.0) | 67.0 (54.0-74.0) | 0.9 |
| Male sex | 317 | 205 / 317 (65%) | 90 / 138 (65%) | 96 / 143 (67%) | 6 / 15 (40%) | 13 / 21 (62%) | 0.2 |
| Body Mass Index | 316 | 26.1 (23.4-29.7) | 26.3 (23.4-29.4) | 25.1 (23.4-29.7) | 29.6 (25.5-33.2) | 26.4 (23.1-31.2) | 0.4 |
| SOFA score | 267 | 6.0 (5.0-8.0) | 6.0 (4.5-7.5) | 6.0 (5.0-8.0) | 6.5 (6.0-9.5) | 9.0 (7.0-10.0) | **0.003** |
| Sub-SOFA respiratory system | 299 | 3.0 (2.0-3.0) | 3.0 (2.0-3.0) | 3.0 (2.0-3.0) | 3.0 (2.0-3.0) | 2.0 (2.0-3.0) | 0.5 |
| Sub-SOFA nervous system | 317 | 0.0 (0.0-1.0) | 0.0 (0.0-1.0) | 0.0 (0.0-1.0) | 0.0 (0.0-1.5) | 1.0 (0.0-1.0) | 0.7 |
| Sub-SOFA cardiovascular system | 310 | 1.0 (0.0-1.0) | 1.0 (0.0-1.0) | 1.0 (0.0-1.0) | 1.0 (1.0-1.0) | 1.0 (1.0-1.0) | 0.2 |
| Sub-SOFA liver | 290 | 0.0 (0.0-1.0) | 0.0 (0.0-0.0) | 0.0 (0.0-1.0) | 0.0 (0.0-0.5) | 0.0 (0.0-2.0) | 0.5 |
| Sub-SOFA coagulation | 316 | 0.0 (0.0-1.0) | 0.0 (0.0-1.0) | 0.0 (0.0-1.0) | 0.0 (0.0-2.0) | 1.0 (0.0-2.0) | **0.02** |
| Sub-SOFA kidneys | 315 | 1.0 (0.0-2.0) | 1.0 (0.0-2.0) | 0.0 (0.0-2.0) | 2.0 (1.0-3.0) | 2.0 (1.0-4.0) | **<0.001** |
| APACHE score | 317 | 18.0 (15.0-22.0) | 18.0 (15.0-21.0) | 17.0 (14.0-21.0) | 21.0 (18.0-24.5) | 21.0 (19.0-24.0) | **0.008** |
| **SIRS criteria** |  |  |  |  |  |  |  |
| Temperature ≤36°C or ≥38°C | 317 | 240 / 317 (76%) | 111 / 138 (80%) | 104 / 143 (73%) | 10 / 15 (67%) | 15 / 21 (71%) | 0.3 |
| Heart rate ≥90 beats/min | 317 | 292 / 317 (92%) | 133 / 138 (96%) | 127 / 143 (89%) | 13 / 15 (87%) | 19 / 21 (90%) | **0.049** |
| Tachypnea, hypocapnia, or mechanical ventilation | 316 | 281 / 316 (89%) | 126 / 138 (91%) | 123 / 143 (86%) | 14 / 15 (93%) | 18 / 20 (90%) | 0.6 |
| Leukocytosis, leukopenia, or left shift |  | 233 / 317 (74%) | 101 / 138 (73%) | 106 / 143 (74%) | 11 / 15 (73%) | 15 / 21 (71%) | >0.9 |
| **Physiological variables *** |  |  |  |  |  |  |  |
| Max. heart rate (1/min) | 316 | 115.0 (101.0-129.0) | 114.5 (102.0-130.0) | 115.0 (100.3-127.0) | 119.0 (99.0-125.5) | 117.0 (103.0-139.0) | 0.9 |
| Min. MAP (mmHg) | 299 | 65.0 (60.0-75.0) | 65.0 (60.0-75.0) | 65.0 (60.0-76.3) | 64.0 (59.5-71.5) | 64.5 (58.0-67.3) | 0.6 |
| Max. respiratory rate (1/min) | 270 | 29.0 (24.0-33.0) | 29.0 (24.0-33.0) | 29.0 (23.3-34.0) | 27.0 (24.0-31.0) | 27.0 (24.3-32.0) | 0.8 |
| Urinary excretion (ml/24h) | 307 | 1,810.0 (1,059.3-3,016.0) | 2,000.0 (1,200.0-3,200.0) | 1,860.0 (1,192.5-2,886.5) | 1,130.0 (784.0-2,784.0) | 945.5 (555.5-1,999.1) | **0.023** |
| Min. PaO_2_/FiO_2_ ratio (mmHg) | 289 | 188.0 (130.0-254.0) | 180.3 (132.3-241.3) | 190.0 (123.0-255.0) | 188.0 (124.3-231.3) | 241.0 (171.5-285.3) | 0.2 |
| **Organ dysfunction** |  |  |  |  |  |  |  |
| Acute encephalopathy^1^ | 315 | 77 / 315 (24%) | 35 / 138 (25%) | 32 / 141 (23%) | 4 / 15 (27%) | 6 / 21 (29%) | 0.9 |
| Renal dysfunction² | 317 | 124 / 317 (39%) | 58 / 138 (42%) | 46 / 143 (32%) | 6 / 15 (40%) | 14 / 21 (67%) | **0.018** |
| Coagulopathy^3^ | 317 | 56 / 317 (18%) | 19 / 138 (14%) | 27 / 143 (19%) | 4 / 15 (27%) | 6 / 21 (29%) | 0.2 |
| Arterial hypoxemia^4^ | 316 | 214 / 316 (68%) | 95 / 137 (69%) | 99 / 143 (69%) | 9 / 15 (60%) | 11 / 21 (52%) | 0.4 |
| Microcirculatory dysfunction^5^ | 316 | 105 / 316 (33%) | 39 / 138 (28%) | 44 / 142 (31%) | 9 / 15 (60%) | 13 / 21 (62%) | **0.003** |
| **Laboratory values** |  |  |  |  |  |  |  |
| Leukocytes min. [G/l] | 314 | 12.0 (7.9-18.0) | 13.0 (8.0-18.9) | 13.0 (8.0-17.0) | 10.0 (6.6-12.0) | 11.0 (7.0-18.0) | 0.2 |
| Platelets, min. [G/l] | 316 | 196.5 (132.8-280.5) | 206.5 (147.3-285.5) | 201.0 (130.5-291.8) | 185.0 (79.5-209.0) | 138.0 (63.0-202.0) | **0.025** |
| Max. bilirubin (µmol/l) | 259 | 12.0 (8.0-21.0) | 14.0 (8.0-20.0) | 12.0 (8.0-21.0) | 15.0 (9.3-24.3) | 14.0 (7.0-55.5) | 0.7 |
| Max. serum creatinine (µmol/l) | 249 | 115.0 (71.0-206.0) | 124.0 (85.0-211.0) | 97.1 (62.0-150.1) | 205.0 (152.6-300.8) | 220.5 (154.7-359.0) | **<0.001** |
| Max. urea (mmol/l) | 303 | 9.5 (6.0-15.0) | 9.1 (6.0-15.0) | 8.0 (5.0-13.0) | 18.1 (13.2-22.5) | 19.5 (12.0-20.3) | **<0.001** |
| CRP level [mg/l] | 240 | 219.0 (138.8-293.0) | 218.0 (139.0-268.2) | 202.0 (131.5-287.4) | 272.0 (234.0-369.5) | 256.0 (210.7-294.8) | 0.2 |
| PCT level [ng/ml] | 227 | 3.0 (1.0-10.0) | 2.0 (1.0-6.0) | 2.0 (1.0-8.0) | 4.0 (2.0-10.0) | 27.0 (15.0-54.5) | **<0.001** |
| Max. lactate at baseline [mmol/l] | 307 | 1.8 (1.2-2.6) | 1.6 (1.1-2.7) | 1.8 (1.3-2.6) | 2.1 (1.7-2.8) | 2.1 (1.1-2.7) | 0.2 |
| bioADM [pg/mL] | 317 | 55.6 (33.4-88.1) | 52.2 (31.8-72.6) | 48.3 (30.4-70.1) | 187.0 (150.9-197.8) | 230.4 (161.7-300.9) | **<0.001** |
| **Focus of infection** | 317 |  |  |  |  |  |  |
| Pneumonia |  | 122 / 317 (38%) | 65 / 138 (47%) | 50 / 143 (35%) | 4 / 15 (27%) | 3 / 21 (14%) | **0.017** |
| Other infections of upper or lower airways |  | 17 / 317 (5.4%) | 8 / 138 (5.8%) | 8 / 143 (5.6%) | 1 / 15 (6.7%) | 0 / 21 (0%) | >0.9 |
| Thoracic (Empyem / Mediastinitis) |  | 8 / 317 (2.5%) | 1 / 138 (0.7%) | 6 / 143 (4.2%) | 0 / 15 (0%) | 1 / 21 (4.8%) | 0.5 |
| Gastrointestinal |  | 14 / 317 (4.4%) | 5 / 138 (3.6%) | 7 / 143 (4.9%) | 0 / 15 (0%) | 2 / 21 (9.5%) | 0.9 |
| Primary bacteremia |  | 4 / 317 (1.3%) | 1 / 138 (0.7%) | 1 / 143 (0.7%) | 1 / 15 (6.7%) | 1 / 21 (4.8%) | 0.3 |
| Catheter-associated infection |  | 8 / 317 (2.5%) | 1 / 138 (0.7%) | 3 / 143 (2.1%) | 1 / 15 (6.7%) | 3 / 21 (14%) | 0.068 |
| Bones/soft tissues |  | 26 / 317 (8.2%) | 9 / 138 (6.5%) | 14 / 143 (9.8%) | 2 / 15 (13%) | 1 / 21 (4.8%) | >0.9 |
| Surgical wound infection |  | 6 / 317 (1.9%) | 2 / 138 (1.4%) | 2 / 143 (1.4%) | 1 / 15 (6.7%) | 1 / 21 (4.8%) | 0.6 |
| Intra-abdominal |  | 59 / 317 (19%) | 20 / 138 (14%) | 33 / 143 (23%) | 4 / 15 (27%) | 2 / 21 (9.5%) | 0.5 |
| Central nervous system |  | 3 / 317 (0.9%) | 1 / 138 (0.7%) | 2 / 143 (1.4%) | 0 / 15 (0%) | 0 / 21 (0%) | >0.9 |
| Genitourinary |  | 38 / 317 (12%) | 16 / 138 (12%) | 13 / 143 (9.1%) | 1 / 15 (6.7%) | 8 / 21 (38%) | 0.051 |
| Unknown source of infection |  | 53 / 317 (17%) | 25 / 138 (18%) | 23 / 143 (16%) | 2 / 15 (13%) | 3 / 21 (14%) | >0.9 |

*^1^* Median (25%-75%); n / N (%)
*^2^* Kruskal-Wallis rank sum test; Pearson's Chi-squared test; Fisher's exact test
Abbrev.: SOFA: Sequential Organ Failure Assessment; APACHE: Acute Physiology And Chronic Health Evaluation ; SAPS: simplified acute physiology score ; SIRS: systemic inflammatory response syndrome; MAP: mean arterial pressure ; PCT: procalcitonin; CRP: C-reactive protein; bio-ADM: bioactive adrenomedullin; ICU, intensive care unit. * 24 h time interval before study inclusion; ¹ reduced vigilance, restlessness, disorientation, delirium, unaffected by psychotropic drugs; ² oliguria: < 0.5 mL/kg/h for at least 2 hours despite adequate volume replacement and/or creatinine increase ≥ 2 times above the reference value of the respective laboratory and/or renal replacement therapy; ³ thrombocytopenia ≤ 100,000/µL or more than a 30% decrease from baseline within 24 hours (not caused by bleeding or immunological factors); ⁴ PaO2 ≤ 75 mmHg [≤ 10 kPa] under room air or a PaO2/FiO2 ≤ 250 mmHg [≤ 33 kPa] under oxygen administration. (Not due to pre-existing heart or lung disease); ⁵ Lactate > 1.5 times the upper reference range and/or base deficit ≥ 5 mmol/L and/or metabolic acidosis with pH < 7.3 and/or impaired capillary reperfusion and/or marbling and/or significant edema in capillary leak syndrome.

**Suppl. Table 10:** Main outcomes of patient population by bio-ADM at cut-off **136 pg/mL** and treatment arm.

| **Outcomes** | **N** | **Overall**, N = 317*^1^* | **<136 Placebo**, N = 138*^1^* | **<136 HC**, N = 143*^1^* | **over 136 Placebo**, N = 15*^1^* | **over 136 HC**, N = 21*^1^* | **p-value***^2^* |
| --- | --- | --- | --- | --- | --- | --- | --- |
| Septic shock within 14 days | 317 | 64 / 317 (20%) | 24 / 138 (17%) | 28 / 143 (20%) | 6 / 15 (40%) | 6 / 21 (29%) | 0.15 |
| 28-day survival | 302 | 289 / 302 (96%) | 128 / 133 (96%) | 131 / 137 (96%) | 14 / 14 (100%) | 16 / 18 (89%) | 0.4 |
| 90-day survival | 284 | 256 / 284 (90%) | 118 / 126 (94%) | 117 / 128 (91%) | 8 / 14 (57%) | 13 / 16 (81%) | **0.001** |
| 180-day survival | 308 | 233 / 308 (76%) | 109 / 134 (81%) | 105 / 139 (76%) | 8 / 15 (53%) | 11 / 20 (55%) | **0.012** |
| ICU length of stay [d] | 315 | 7.0 (4.5-15.0) | 7.0 (5.0-15.0) | 7.0 (4.0-12.0) | 16.0 (10.0-23.0) | 7.0 (6.0-37.0) | **0.027** |
| Hospital length of stay [d] | 315 | 22.0 (14.0-44.0) | 19.0 (13.0-33.0) | 22.0 (14.0-42.0) | 51.0 (39.0-181.0) | 44.0 (24.0-181.0) | **<0.001** |
| Days with mech ventilation until D28 (on ICU) | 315 | 1.0 (0.0-6.0) | 2.0 (0.0-6.0) | 1.0 (0.0-5.0) | 7.0 (1.0-13.5) | 2.0 (0.0-9.0) | 0.2 |
| Δ SOFA score | 246 | 1.0 (-1.0-3.0) | 0.0 (-1.0-3.0) | 0.5 (-1.0-2.0) | 2.0 (1.0-3.8) | 3.0 (1.0-5.0) | **0.018** |
| Days with RRT until D28 (on ICU)*^3^* | 315 | 0.7 (2.7) | 0.7 (2.9) | 0.5 (2.4) | 0.5 (1.1) | 2.3 (4.1) | **0.004** |

*^1^*Median (25%-75%); n / N (%)

*^2^*Wilcoxon rank sum test; Pearson's Chi-squared test
*^3^*Mean (sd); Median and interquartile range are 0.
